# Benefits and Limitations Associated with the Use of Alternative Biological Matrices in Forensic Toxicology, as Well as Genetic Markers of Poisoning and Psychoactive Substance Abuse

**DOI:** 10.3390/ijms27167212

**Published:** 2026-08-12

**Authors:** Aleksandra Zorychta, Marcin Tomsia, Rafał Skowronek, Elżbieta Chełmecka

**Affiliations:** 1 Department of Medical Statistics, Faculty of Pharmaceutical Sciences, Medical University of Silesia, Ostrogórska 30, 41-200 Sosnowiec, Poland; aleksandra.zorychta@sum.edu.pl; 2 Department of Forensic Medicine and Forensic Toxicology, Faculty of Medical Sciences (FOMS) in Katowice, Medical University of Silesia, 18 Medyków Street, 40-752 Katowice, Poland; mtomsia@sum.edu.pl

**Keywords:** post mortem, alternative matrices, forensic toxicology, toxicology, drugs, forensic epigenetics

## Abstract

In forensic toxicology, the analysis of blood and urine is regarded as the so-called “gold standard.” However, when forensic experts are confronted with advanced postmortem changes, it becomes necessary to secure and examine alternative matrices. Suitable specimens in cases of advanced putrefactive decomposition may include: skin appendages (hair and nails), bones, bone marrow, cartilage tissue, teeth, antemortem fingerprints, cerebrospinal fluid, vitreous humor, breast milk, meconium, placenta and umbilical cord tissue, oral fluid, sweat, and other evidentiary materials. These tissues, owing to their structure and anatomical location, are more resistant to putrefactive decomposition than body fluids and soft tissues. However, several limitations complicate the reliable analysis of these matrices. These include incomplete drug incorporation, depending on physicochemical properties, the inability to correlate analyte concentrations with pharmacological effects, low xenobiotic levels, and the need for highly sensitive analytical methods. Collectively, these factors contribute to interpretative challenges during forensic expert evaluation and reporting. To address key forensic questions, epigenetic variability analyses (forensic epigenetics) may also be employed, particularly when standard DNA profiling is uninformative. DNA methylation patterns in specific tissues and in individual subjects can be used, among other purposes, to identify the tissue of origin of a human biological trace, to differentiate monozygotic twins, and to predict the age of an unidentified trace donor. Over the past few years, this approach has gained increasing importance; in the context of forensic trace analysis, it offers both advantages and limitations. The aim of this study is to present current scientific evidence regarding the use of alternative biological matrices as potential sources of information of forensic relevance. The paper discusses the characteristics of individual biological materials, with particular emphasis on their analytical properties, potential applications, limitations, and possible advantages in forensic toxicological investigations. Furthermore, the key aspects associated with the application of epigenetic methods in forensic science are presented, with particular focus on their role in individual identification and the reconstruction of circumstances surrounding forensic events. Alternative biological matrices and modern analytical approaches may constitute valuable complementary tools to conventional evidentiary materials used in forensic medicine and criminalistics. Due to their physicochemical properties, stability, and ability to preserve specific biological information, these matrices may provide significant data that enable substance identification, assessment of exposure to psychoactive compounds, reconstruction of event circumstances, and support for identification procedures. However, their application requires consideration of analytical limitations, the specific characteristics of the examined matrix, and the necessity for standardization and validation of diagnostic procedures

## 1. Introduction

Over the past several decades, forensic toxicology has undergone rapid development. As a forensic science discipline, it has broad applications in cases of various types, including drug-related deaths, matters involving the use of doping agents, and investigations concerning road traffic safety [1]. An important area of toxicology application includes the analysis of cases aimed at distinguishing accidental poisonings from intentional acts of a criminal nature. Comprehensive knowledge of the mechanisms of action of toxic substances, their pharmacokinetic properties, and toxicological potential may be used both in the diagnosis of poisoning cases and in reconstructing events associated with exposure to harmful agents. The high toxicity of certain compounds and their short persistence in the body may represent significant limitations in the detection process; however, advances in toxicological research and the implementation of modern analytical techniques enable increasingly precise identification of substances that are otherwise difficult to detect [2,3].

A particularly important aspect in this context is the forensic application of veterinary toxicology, which includes the investigation of cases involving intentional animal poisoning. The literature describes situations in which the poisoning of animals under the shared care of individuals involved in interpersonal conflicts constituted an element of retaliatory or criminal actions. Such cases require an interdisciplinary approach integrating knowledge from toxicology, veterinary medicine, and forensic science [4].

The results of toxicological analyses provide a wealth of important information. At the moment of death, all metabolic processes involving drugs and other substances cease. If the autopsy is performed promptly and the body has not been exposed to adverse environmental conditions, forensic toxicological testing constitutes a key source of evidence. It makes it possible to determine which substances or drugs were present in the body at the time of death. Quantitative analysis, in turn, can indicate whether an overdose of medications or other substances occurred. Particularly valuable information includes assessment of sobriety (blood ethanol concentration) and whether the deceased was under the influence of other psychoactive substances at the time of death (drugs of abuse, medicines) [5].

Detailed recommendations regarding sample collection have been established by TIAFT (The International Association of Forensic Toxicologists)—an organization of major importance for the success of Systematic Toxicological Analysis (STA). The accuracy and reliability of every result are closely linked to compliance with guidelines, including those concerning sample collection (type of specimen, the amount to be submitted to the laboratory, and the quality of collection). STA is largely responsible for selecting an appropriate analytical strategy—one aimed at detecting potentially toxic compounds and their metabolites in biological samples. Therefore, it is crucial to provide the toxicologist with the best-preserved specimen possible, along with the case history relevant to the investigation. When an unknown substance or an unknown drug may be involved, reliable toxicological examinations are required, strictly following STA recommendations [6].

**Figure 1:**
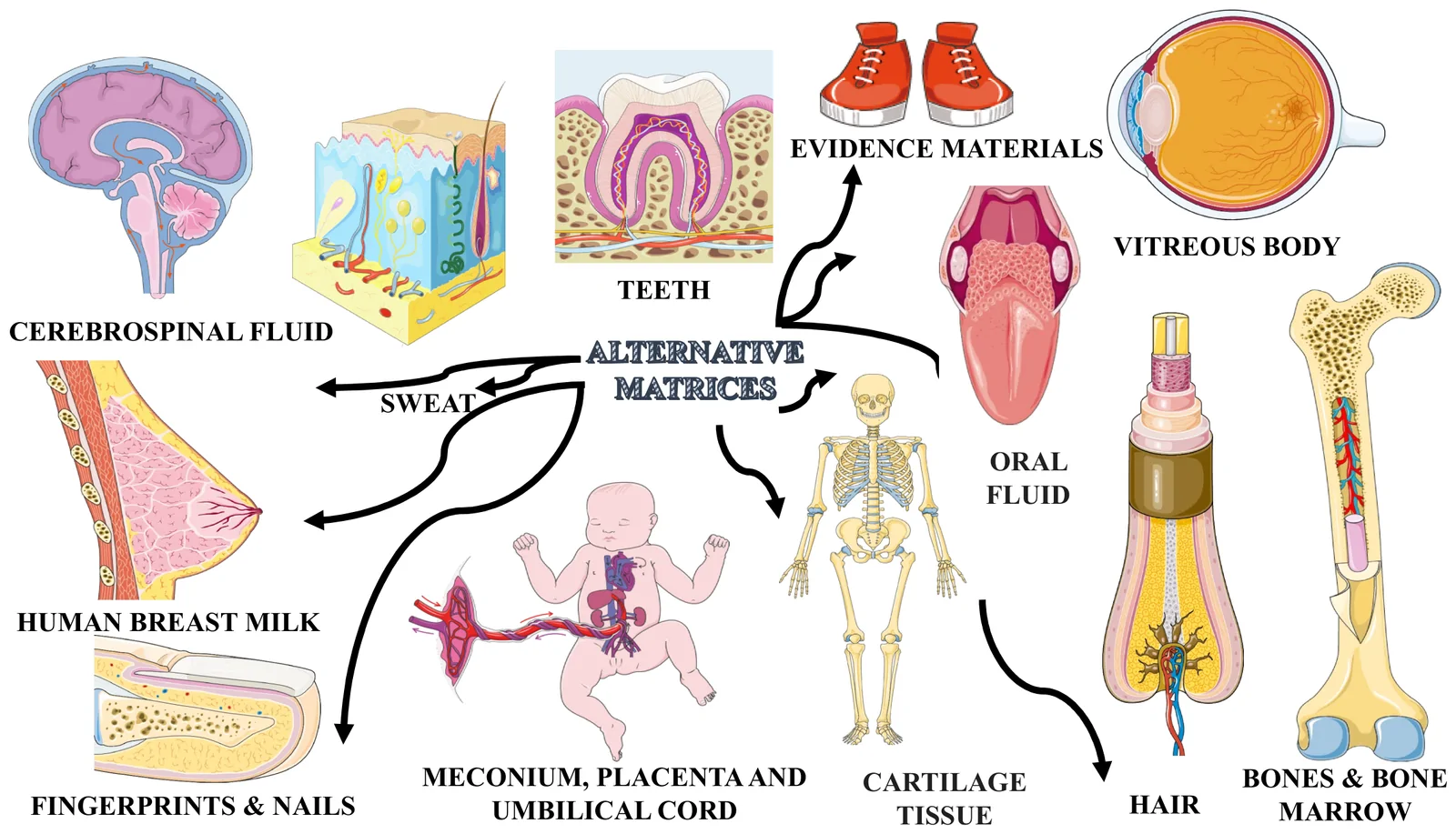
Types of alternative matrices prepared using the smart.servier.com website. Created by the authors using SMART—Servier Medical ART. Medical illustrations are provided by Servier Medical ART (SMART) and are licensed under the Creative Commons Attribution 4.0 International License (CC BY 4.0).

In forensic toxicology, blood and urine are considered the gold standard analytical matrices [7,8]. In bodies undergoing advanced putrefactive decomposition or in skeletonized human remains, conventional matrices may no longer be available for analysis. Therefore, during medicolegal autopsy, alternative matrices are collected, such as skin appendages (hair and nails), bones, bone marrow, cartilage tissue, teeth, cerebrospinal fluid, vitreous humor, meconium, placenta, and umbilical cord tissue. In addition to autopsy materials, alternative matrices can also provide important information, including drug use fingerprints, breast milk, saliva, and sweat [8,9,10]. Besides both autopsy and non-autopsy materials, other types of physical evidence may also constitute an important source of information. The above-mentioned alternative matrices and evidentiary materials are presented in Figure 1.

A highly significant phenomenon throughout the entire post-mortem analytical process is post-mortem redistribution (PMR). Over the years, it has come to be referred to as the “toxicologists’ nightmare” and as a phenomenon of “dramatic extent in the post-mortem setting.” [11]. Post-mortem redistribution refers to a range of anatomical and physiological changes occurring after death that may lead to spurious drug concentration results. Redistribution occurs predominantly via diffusion of drugs along the concentration gradient (from sites of high concentration in solid organs into the blood), thereby contributing to falsely elevated blood drug concentrations. Redistribution occurs in the first post-mortem hours, just before the onset of putrefaction. Immediately after death, major biochemical changes take place; among other things, the sodium–potassium pump (Na^+^/K^+^-ATPase) ceases to function, which contributes to a sudden increase in potassium concentration and to destabilization of the cell membrane. Consequently, metabolism is inhibited, and complex transport mechanisms are also suppressed. Susceptibility to PMR is assessed by comparing the amount (concentration) of an analyte in central blood with that in peripheral blood—the cardiac-to-peripheral blood concentration ratio (C/P). Because cardiac (central) blood is more susceptible to PMR-related changes than peripheral blood, the C/P ratio is widely used in forensic medicine as a marker for assessing PMR. Long-term research on PMR has led to the identification of nine main mechanisms, including: passive diffusion (e.g., from the lungs, liver, heart, and gastrointestinal tract), post-mortem body changes (e.g., blood hypostasis, blood movement, cellular death, and putrefactive processes), and the chemical and pharmacokinetic properties of the analyte [11,12].

Table 1 presents a summary of the most important classified PMR mechanisms together with their characteristics.

Almost all drugs exhibit some degree of susceptibility to PMR; however, the greatest propensity is observed for drugs that are basic, lipophilic, highly protein-bound, and characterized by a large Vd. Substantial evidence indicates that postmortem redistribution (PMR) occurs predominantly in basic drugs with high lipophilicity and a large volume of distribution. However, these physicochemical properties alone do not fully explain the redistribution observed in certain therapeutic agents that are neither basic nor highly lipophilic [14].

The postmortem interval, body position, and route of drug administration are among the most important factors influencing postmortem changes in drug concentrations. Whenever possible, femoral blood should be collected as soon as possible after death for toxicological analysis, as it is generally less susceptible to PMR than central blood [11].

Nevertheless, available studies have demonstrated that femoral blood is not invariably protected from postmortem changes. Therefore, the analysis of blood collected from multiple sampling sites is recommended, particularly in individuals receiving chronic methadone therapy [17].

**Figure 2:**
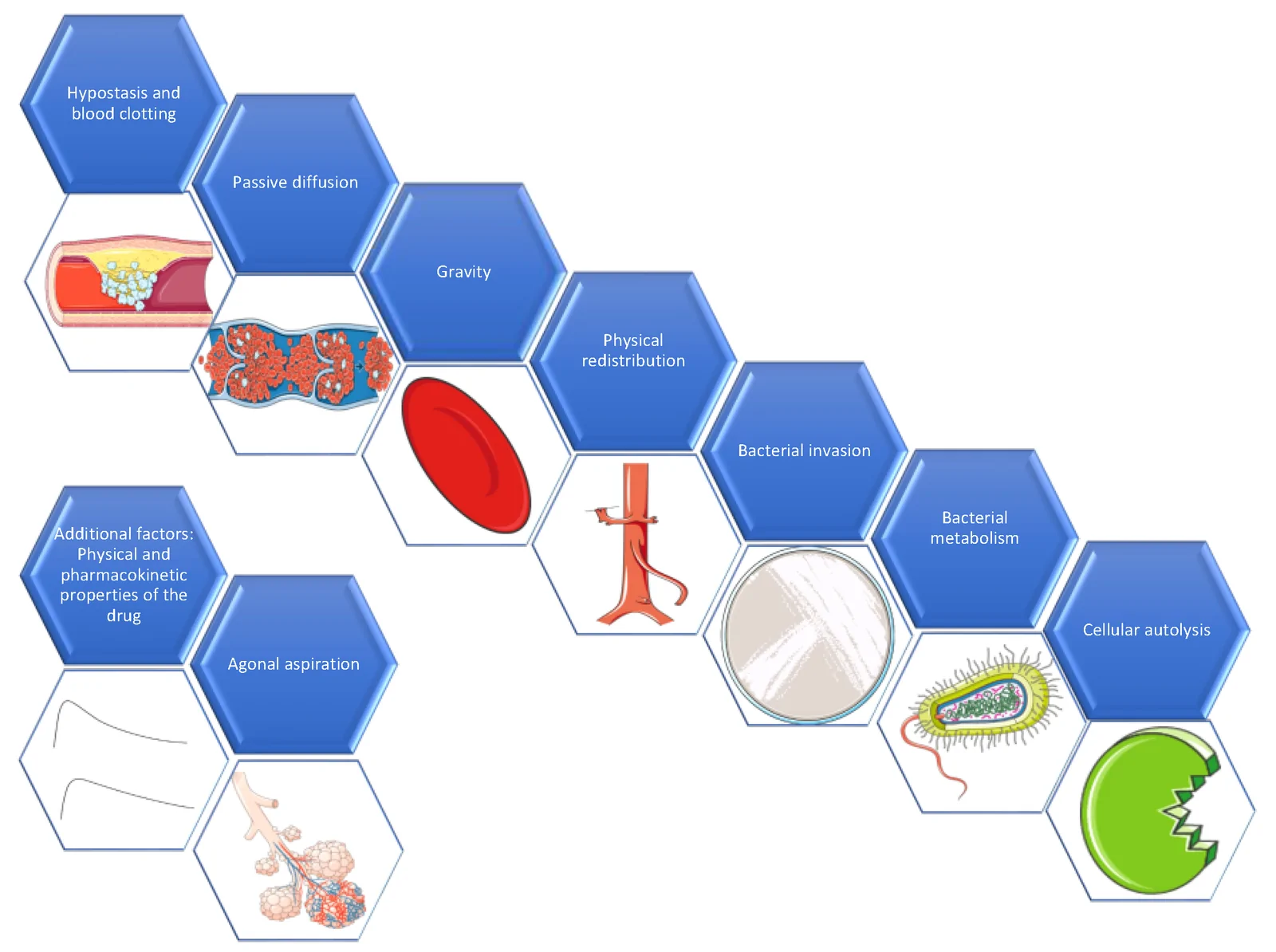
Types of postmortem redistribution (PMR) mechanisms prepared using the smart.servier.com website. Created by the authors using SMART—Servier Medical ART. Medical illustrations are provided by Servier Medical ART (SMART) and are licensed under the Creative Commons Attribution 4.0 International License (CC BY 4.0).

All available information regarding the mechanisms of PMR, together with a detailed analysis of physiological changes occurring in the body during both the early and advanced postmortem stages, assists forensic pathologists in estimating the postmortem interval (PMI) (Figure 2).

Additionally, forensic entomological analysis is frequently performed as a complementary approach to support and expand the available evidence. Information concerning insect life cycles and the species responsible for colonization of human remains may provide valuable data for the estimation of PMI [18].

The concept of the thanatomicrobiome deserves particular attention in the estimation of the PMI. This term refers to the analysis of microorganisms distributed throughout internal organs and external body openings after death. Recent findings indicate that anaerobic bacteria of the genus *Clostridium* spp. are among the predominant microorganisms colonizing the human body after death. Before death, microbiome-associated cells are estimated to account for approximately 75–90% of the total cellular content of the human body.

Studies have shown that information regarding the abundance and distribution of specific microorganisms within individual organs may, in the future, be applied as a valuable tool for estimating the PMI [19,20].

Using sequencing-based methods, a group of researchers identified six bacterial taxa originating from partially skeletonized lower rib bones. As the bones gradually transitioned into a dry skeletal state, the microbial communities became increasingly similar to those found in soil environments. It has been suggested that the inhibition of bacterial proliferation during the early stages of skeletonization may be associated with the preservation of organic compounds contained within the bone tissue [21]. The lack of reliable methods for estimating the PMI in soft tissues and bone has contributed to increased interest in, and a better understanding of, the mechanisms underlying skeletal decomposition. Current methods are not only of limited reliability but are also time-consuming, costly, and unsuitable for cases involving a PMI exceeding 5 years.

Raman spectroscopy (RS) has the potential to serve as an innovative and non-destructive method in forensic medicine for PMI estimation. This approach enables PMI assessment through the analysis of time-dependent chemical modifications occurring in bones and teeth, correlated with the duration of burial [22].

Chemical changes occurring in human remains continue to be regarded as one of the most promising approaches for PMI estimation. Proteomics enables the identification of differences in the biochemical properties of a wide range of proteins and the quantitative assessment of individual proteins. These capabilities support the identification of potential biomarkers of the postmortem interval (PMI) [23].

## 2. Alternative Matrices

### 2.1. Hair

Keratinized matrices, such as hair, are capable of stably accumulating certain drugs, thereby providing information regarding exposure to various substances during the period preceding death. For this reason, hair has considerable forensic and clinical significance [24].

Human hair is frequently used as a biological matrix in forensic toxicology because it provides valuable information on an individual’s history of substance exposure. Based on the rate of hair growth, it may also be used to estimate the duration of drug use or pharmacotherapy. Analytical techniques commonly applied for the detection of toxic substances include high-performance liquid chromatography (HPLC), gas chromatography–mass spectrometry (GC–MS), liquid chromatography–tandem mass spectrometry (LC–MS/MS), and gas chromatography–tandem mass spectrometry (GC–MS/MS). These methods enable the detection of a wide range of compounds, including ketamine, methamphetamine, and amphetamine, in hair samples [25,26,27,28,29].

The choice of analytical method depends on several factors, including the purpose of the examination, the analytical characteristics of the target compound, method feasibility, and the availability of analytical instrumentation [30].

The actual time frame of drug incorporation into a hair sample is determined based on hair length and the relative contribution of individual phases of the hair growth cycle. Two main types of hair are distinguished: anagen hair (actively growing) and catagen and telogen hair (resting phases). Through segmental analysis (i.e., examination of hair along its longitudinal axis), long-term monitoring of toxic substance exposure is possible, even following a single administration of the substance [31,32,33].

Excluding analytical errors, false-positive results in hair analysis may occur due to contamination caused by environmental exposure (so-called external contamination). Another potential source of misleading results is the incorporation of drugs into hair from various biological fluids, such as sweat or putrefactive fluids, which may represent a postmortem artifact.

Kinz P. emphasized that even the most rigorously performed standard decontamination procedures are unable to eliminate external contamination in postmortem hair samples [34].

Particular attention in hair sample analysis should be given to gamma-hydroxybutyric acid (GHB). Endogenous GHB is a potent central nervous system depressant. Before its withdrawal from medical use in the United States in 1990, it was applied, among other indications, as an anesthetic agent and in obstetrics as a cervical ripening agent. During the 1980s, GHB was widely available in health food stores, as it was believed to promote reduction in adipose tissue through the release of growth hormone, making it a popular substance among bodybuilders.

Currently, GHB, commonly referred to as the “date rape drug,” is associated with criminal activity and is used in cases involving drug-facilitated crime. Due to its very short half-life, detection of GHB in blood and urine samples is extremely challenging. In such cases, hair analysis, owing to its extended detection window, represents a valuable and, in certain circumstances, the only available source of toxicological evidence [25,28,35].

Zolpidem has also been recognized as a substance used in drug-facilitated sexual assault. Substances administered in cases of sexual assault are often difficult to detect in blood and urine samples due to the low doses involved and their rapid elimination from biological fluids. In such cases, there is frequently a significant delay between the occurrence of the incident and its reporting to law enforcement authorities. Moreover, the amnestic effects of these substances may further complicate the accurate recollection and reporting of events [36].

### 2.2. Nails

Fingernails, as keratinized matrices similar to hair, are increasingly being used as complementary analytical matrices in forensic and clinical toxicology. The primary mechanism of drug incorporation into nails occurs during nail plate formation within the germinal matrix.

The literature describes three main pathways of drug incorporation into the nail matrix. The individual mechanisms of incorporation, together with their respective pathways, are presented in Table 2.

Nail analysis is of particular importance for substances exhibiting specific properties that allow their long-term accumulation within the nail matrix [37,38,39].

Studies indicate that fingernail clippings may enable retrospective assessment of substance exposure for up to 5 months prior to sample collection, whereas toenail clippings may provide information on exposure periods extending up to 14 months before collection [40]. In the majority of comparative studies, concentrations of the analyzed substances were lower in fingernails than in toenails [41].

The mechanisms underlying drug incorporation into nails remain incompletely understood. Moreover, substantial interindividual variability related to physiological, biochemical, and pharmacokinetic factors may contribute to inaccurate quantitative drug analysis.

In elderly individuals and patients with hepatic or renal impairment, reduced drug metabolism and elimination may occur, potentially resulting in elevated concentrations of the analyzed substance. Sex-related differences may also influence the assessment of drug absorption, distribution, metabolism, and excretion.

Therefore, prolonged drug use should not be assumed to correlate directly with higher concentrations of the substance in the analyzed sample [24].

### 2.3. Bones

In cases of complete skeletonization, bones represent the primary and often the only source of toxicological information [10,42,43]. Bone tissue and bone marrow may account for approximately 14% of total body mass. Following the decomposition of skin and soft tissues, human remains undergo skeletonization, resulting in complete exposure of bones to the external environment.

Although bone is more resistant to degradation than soft tissue, it is not entirely stable. Over time, bone tissue undergoes progressive deterioration. After death, bones are subject to both decomposition and diagenesis, defined as postmortem alterations occurring after burial or environmental exposure. The factors contributing to these changes include chemical, physical, and biological processes, which may alter the original composition of the bone matrix [44].

Microbial colonization of bone is an important mechanism contributing to postmortem skeletal degradation. When hydroxyapatite, one of the primary components of bone, loses equilibrium with the external environment, recrystallization occurs, accelerating the degradation of this potential toxicological matrix.

Dissolution of the bioapatite phase exposes the organic matrix, the second major component of bone, thereby facilitating microbial and enzymatic activity involved in further decomposition processes [44,45].

The main advantage of bone as a postmortem toxicological matrix, compared with other alternative tissues, is its protected anatomical location. This characteristic significantly reduces the risk of contamination by exogenous substances [46].

Bone tissue is unique in its ability to preserve active substances for extended periods after death. Studies have shown that toxicological compounds may remain detectable in bone tissue for up to 23 years postmortem [47,48].

Gas chromatography–mass spectrometry (GC–MS) is a widely used and validated analytical method for the detection of toxicological substances in bone tissue. This technique has been successfully applied for the identification and quantification of various compounds in human bones, including antidepressants (venlafaxine, amitriptyline, and duloxetine), opioids (6-monoacetylmorphine, morphine, codeine, methadone, tramadol, and cocaine), and benzodiazepines (nordiazepam, oxazepam, lormetazepam, lorazepam, clonazepam, bromazepam, and alprazolam) [49,50,51,52].

The second analytical technique applied for bone tissue examination is liquid chromatography–tandem mass spectrometry (LC–MS/MS). This method has been validated for the determination of benzodiazepines and antidepressants in bone samples [53].

The use of LC–MS/MS in forensic science has increased significantly. Initially, this technique was applied less frequently and mainly served as an alternative to GC–MS for the analysis of challenging compounds, including thermolabile substances and highly polar analytes.

Currently, LC–MS/MS is among the most widely used analytical techniques in forensic toxicology. Its ability to provide structural information, combined with high sensitivity, makes it a valuable tool in forensic investigations.

Unlike GC–MS, LC–MS/MS does not require derivatization, a process involving the conversion of an analyte into derivatives with physicochemical properties that facilitate analytical detection and quantification [54].

For example, the analysis of benzodiazepines, which are non-volatile and polar compounds, by gas chromatography (GC) and GC–MS is highly challenging. Therefore, high-performance liquid chromatography (HPLC) is commonly used for their detection [55].

### 2.4. Bone Marrow

Bone marrow is a structurally and functionally diverse connective tissue, with a total mass of approximately 3 kg in an adult human body. Its chemical composition consists primarily of water, lipids, and proteins.

Depending on the predominance of its major components, bone marrow can be classified into two types:

**Red (hematopoietic) bone marrow**—contains erythroid, lymphoid, and myeloid cell lineages, which collectively contribute to hematopoiesis.

**Yellow (fatty) bone marrow**—characterized by the presence of adipocytes, which may regulate the activity of other cell populations within the bone marrow, including bone-forming and hematopoietic cells, functioning as a “critical microenvironmental regulator of bone marrow”.

The remaining components of bone marrow include nerve fibers and blood vessels [56,57].

In newborns, bone marrow is predominantly present as red (hematopoietic) marrow. With age, red marrow gradually undergoes conversion into yellow (fatty) marrow, resulting in a composition of approximately 40% water and 40% fat in red marrow, and 15% water and 80% fat in yellow marrow [58].

Bone marrow performs several essential functions, including hematopoiesis, which involves the production and differentiation of blood cellular components. It also serves as a reservoir of mesenchymal stem cells, which are crucial for skeletal remodeling.

Adipose tissue is an important structural component of bone marrow involved in maintaining homeostasis. For many years, it was considered only a passive space-filling element [59].

The high degree of vascularization and the lipid-rich matrix make bone marrow a potential reservoir for the accumulation of tissue-derived substances. Moreover, its location within the medullary cavity and surrounding bone tissue provide a natural physical barrier that reduces the risk of contamination by external factors, including soft tissue decomposition products and substances present in soil. This anatomical protection also limits postmortem exposure to bacteria, fungi, plants, and animals.

Due to these protective properties, bone marrow is considered one of the most protected tissues in the human body. Consequently, despite its extensive vascularization, postmortem decomposition is significantly delayed [57]. Bone marrow is also protected from chemical exposure, including formaldehyde used during thanatopraxy procedures [60].

Despite these protective features, bone marrow as an analytical matrix is susceptible to various processes that may interfere with, or prevent, the correct interpretation of analytical results. These processes include:

**Postmortem redistribution**

**Evaporation**

**Degradation**

**Neoformation**

The aforementioned processes, together with limited pharmacokinetic data, prevent bone marrow from being considered a standard biological matrix, such as blood or urine. Instead, it is classified as an alternative matrix, primarily used in cases involving advanced decomposition.

Reliable analysis is further complicated by environmental factors, including temperature, humidity, and burial conditions [57,61].

According to available literature data, bone marrow preserved under controlled conditions may remain suitable for toxicological analysis for several years [62]. However, its estimated histological preservation period is considerably shorter, lasting approximately 3 months [57].

Antonio Laudani et al. conducted a study aimed at developing and validating an analytical method for the extraction and determination of various antidepressant and antipsychotic drugs from porcine ribs, with particular emphasis on bone marrow analysis.

Method validation included the assessment of linearity, precision, sensitivity, accuracy, and matrix effects. The obtained results supported the potential application of this method in forensic toxicology protocols as an alternative to conventional analytical procedures [61].

### 2.5. Cartilage Tissue

Cartilage tissue is a flexible connective tissue composed of chondrocytes and characterized by low oxygen and nutrient requirements, as well as relatively low metabolic activity. The surrounding extracellular matrix contains nutrients and other components that diffuse into chondrocytes, supporting their survival for several days after death.

The prolonged postmortem viability of chondrocytes, combined with their gradual decline in response to time and temperature changes, suggests that cartilage tissue may represent a potential future matrix for postmortem interval (PMI) estimation in forensic investigations [63].

Additionally, due to its structural properties, cartilage tissue may serve as an alternative matrix for the postmortem analysis of methanol. Studies assessing methanol concentrations in epiglottic cartilage, costal cartilage, and intervertebral disc cartilage demonstrated correlations with methanol concentrations measured in femoral blood samples.

This finding is of particular importance in cases of fatal intoxication, where conventional biological matrices, such as blood and urine, are no longer available for diagnostic purposes or have been affected by PMR [64].

A similar study was conducted to determine ethanol concentrations using gas chromatography with flame ionization detection (GC–FID). The results obtained from costal cartilage samples also demonstrated correlations with ethanol concentrations measured in femoral blood and urine [65].

Cartilage tissue has additionally been used for the detection of sodium nitrite (NaNO_2_) in a sample obtained from a fatal suicide case, yielding satisfactory results and indicating its potential future application as an alternative forensic matrix in cases of NaNO_2_ poisoning, which is a quite new phenomenon in suicidology [66,67].

Besides PMI estimation and toxicological investigations, cartilage tissue may also be utilized for age estimation [68,69].

Research on the application of cartilage tissue as a matrix in forensic medicine is still ongoing and continues to be further developed and refined.

### 2.6. Teeth

In cases where routine biological matrices, such as blood, urine, or hair, are unavailable, or as an alternative to oral fluid analysis, the examination of hard dental tissues may provide valuable information. Following the administration of a drug, the substance may diffuse into oral fluid, which is subsequently incorporated into the enamel during the process of remineralization.

Over the past several years, studies have been conducted using dental tissues for the detection of various compounds, including drugs of abuse, metals, alcohol, and nicotine [70].

Additional information may also be obtained from dental plaque, defined as a non-mineralized dental biofilm, which has considerable potential as an alternative matrix in forensic toxicology. The composition and structure of dental plaque are heterogeneous, with a diverse population of bacterial species.

The complex architectural organization of dental plaque may contribute to prolonged retention of substances incorporated into its matrix. The extracellular matrix of subgingival dental plaque consists of a complex mixture of macromolecules, the composition and functions of which remain incompletely understood [71].

Drugs are incorporated into dental plaque through direct contact following oral or intranasal administration, resulting in high concentrations of these substances within the oral cavity. The second, indirect pathway involves the incorporation of drugs into dental plaque from secreted saliva, which acts as a filtrate of blood-derived substances.

To evaluate the suitability of dental plaque as an analytical matrix, studies have been conducted using LC–MS/MS following acetonitrile-based extraction for the detection of substances including methadone, morphine, and their metabolites [72,73].

A positive detection of drugs of abuse in hard dental tissue may indicate previous substance use, whereas their detection in dental biofilm may reflect more recent drug administration. The simultaneous use of both matrices—hard dental tissue and dental biofilm—for analytical purposes provides an extended detection window.

A limiting factor affecting drug incorporation into tooth enamel is the formation of dental deposits, which may interfere with substance penetration into the enamel structure [70].

The absence of dental tissue may occur due to edentulism or as a result of environmental factors. Therefore, when available, dental tissue may be utilized as an alternative matrix in forensic toxicology analyses.

When interpreting analytical results, the routes and rates of absorption of different drugs, as well as the physicochemical differences between the tooth root, tooth crown, and carious material, should be taken into consideration.

Studies conducted to detect drugs of abuse and psychoactive substances, including amphetamine, 3,4-methylenedioxymethamphetamine (MDMA), morphine, codeine, norcodeine, methadone, fentanyl, tramadol, diazepam, nordiazepam, and promethazine, demonstrated concentrations consistent with those obtained from biological fluids and hair samples [74].

For the assessment of previous chronic drug consumption, analytical methods based on GC–MS are used [75].

Postmortem tooth discoloration may provide investigators with valuable information useful for narrowing the estimated time of death and assessing the potential cause of death. The phenomenon of postmortem pink teeth refers to a change in dentin coloration resulting from increased intracranial blood pressure, which may potentially lead to hemorrhage within the pulp chamber, the region containing dental blood vessels and nerves.

This phenomenon has been described in a case involving the death of a man who remained submerged in water for approximately one month. The environmental conditions surrounding the deceased (prolonged water exposure) and the position of the body contributed to the development of the pink discoloration, assisting forensic experts in narrowing the postmortem interval and suggesting drowning as the probable cause of death.

Observations such as tooth discoloration may provide important information regarding the circumstances surrounding death; however, currently, there is no definitive evidence confirming a direct association between the pink discoloration of teeth and the cause of death [76,77].

A key element in the identification of unknown human remains is the estimation of the decedent’s age at the time of death. For this purpose, numerous studies have been conducted, including the pulp/tooth area ratio (PAR) method, which determines the size of the pulp cavity as a marker of secondary dentin deposition for age assessment in adults.

Research involving the PAR method has demonstrated that it is a reliable and sex-independent technique, enabling the estimation of age at the time of death [78]. In addition to the aforementioned method, one of the most useful chemical approaches for age estimation is the analysis of aspartic acid racemization (AAR), which is based on the gradual increase in the proportion of the D-enantiomer of aspartic acid in tissues with advancing age [79].

### 2.7. Fingerprints

Fingerprints represent a highly important form of evidence in the field of forensic science worldwide. The most commonly used analytical approach is dactyloscopy, which is based on the comparison of two friction ridge impressions collected from a crime scene with a reference print obtained from an individual.

Personal identification through fingerprint analysis relies on two fundamental characteristics: uniqueness and permanence. In forensic investigations, fingerprints not only contribute to the identification of perpetrators but also have significant potential as an alternative matrix in forensic toxicology.

Fingerprints primarily consist of sweat and sebum deposited on the skin surface, with their composition comprising approximately 95–99% water along with organic components. Substances are transported to the skin surface through sweat via various mechanisms, including passive diffusion from the bloodstream into sweat glands.

The skin of the palms contains exclusively eccrine sweat glands; however, due to frequent contact of the hands with other body regions, traces of sweat originating from sebaceous and apocrine glands may also be present on the surface of the hands [80].

Due to various degradation and oxidation processes, the composition of fingerprints is not constant. This instability results from changes occurring at the interface between the fingerprint residue and the contacted surface.

With increasing time after deposition, fingerprint constituents can be divided into two groups:

compounds originating from the initial composition deposited on the surface;

original compounds together with their degradation products.

Factors influencing the initial composition of sweat may include:

**Donor-related factors** (individual propensity or lack thereof for sebaceous and sweat secretion);

**Type of deposition surface**;

**Environmental factors**;

**Airflow (air circulation)**;

**Contaminants (e.g., dust)**;

**Atmospheric conditions**, including humidity, ultraviolet (UV) radiation, and temperature [81].

Studies conducted using LC–MS/MS have demonstrated that even single drug use events can be detected from fingerprint samples. The detection of drugs in fingerprint residues may be useful for determining whether an individual who touched a particular object was under the influence of a substance at the time of the committed offense.

Another well-established and extensively described approach is the identification of amino acids, as excretory products, for the enhancement and visualization of fingerprints [81,82].

The assessment of fingerprint age remains relatively unreliable at present due to the limited number of studies conducted in this area. Emerging analytical approaches are primarily focused on the physical and chemical changes occurring in fingerprint residues over time, as well as on the analysis of newly formed degradation products and metabolites [83,84,85].

### 2.8. Cerebrospinal Fluid (CSF)

In forensic toxicology, cerebrospinal fluid (CSF) may also serve as an alternative biological matrix when primary biological fluids are unavailable. CSF has a composition similar to that of plasma, with the main difference being the absence of high-molecular-weight proteins.

CSF may be collected either antemortem or postmortem, in accordance with local national guidelines, which should be continuously reviewed and updated.

In Poland, the Polish Society of Forensic Medicine and Criminology (PTMSiK) is an organization responsible for, among other activities, the publication of the guidelines entitled *“Recommendations for the Collection of Postmortem Material for Toxicological Examination”* [86,87]. When the body is in an advanced stage of decomposition, collected CSF may represent a more reliable alternative to blood samples for analytical purposes. The deep anatomical location of CSF within the spinal canal significantly contributes to the reliability of sample analysis.

In studies concerning ethanol intoxication, a strong positive correlation was observed between ethanol concentrations in blood and CSF (Pearson correlation coefficient: *p* < 0.001, *r* = 0.9503) [88].

For postmortem examination purposes, in addition to conventional autopsy, supplementary analysis in the form of postmortem imaging may be performed. This approach includes, among others, the use of magnetic resonance imaging (MRI) and computed tomography (CT) technologies.

Postmortem imaging offers numerous advantages, including the non-invasive nature of the technique and its reproducibility, allowing continuous access to the acquired data. These data may serve as a valuable tool for determining the cause and manner of death [89].

Due to the complexity of pre-analytical CSF assessment and the lack of sufficiently advanced analytical techniques, the potential use of CSF as an alternative matrix in postmortem toxicological investigations, including the detection of narcotic and illicit substances, remains difficult to determine. Despite the increasing interest in postmortem cerebrospinal fluid analysis, the results reported by different authors remain inconsistent and cannot be directly compared [87,90].

### 2.9. Vitreous Body

The vitreous humor is a widely used alternative matrix in postmortem toxicological investigations. In forensic toxicology, analyses are most commonly performed for the detection of, among others, pharmaceuticals, drugs of abuse, and volatile substances [91].

The vitreous humor is a transparent, gelatinous matrix composed primarily of water (approximately 99%) and fibrous proteins, including collagen and hyaluronic acid [92].

Similar to other biological matrices, the vitreous humor has both advantages and limitations. Its major advantages include resistance to postmortem autolysis and the endogenous synthesis of compounds. It demonstrates prolonged stability, which allows an extended time frame for sample collection and analysis.

Furthermore, its anatomical location provides effective protection against decomposition processes, external contamination, and microbial activity, making it a well-preserved matrix for postmortem toxicological investigations.

It contains only a limited number of enzymes that may be present in other tissues (e.g., pseudocholinesterase), making it an excellent matrix for the analysis of substances characterized by rapid metabolism, such as cocaine [93]. Through mechanisms such as diffusion, osmotic pressure, hydrostatic pressure, and active transport, substances can readily pass from the bloodstream across the so-called blood–retinal barrier (BRB) [94]. The penetration of substances is also influenced by their physicochemical and pharmacokinetic properties, as well as by the condition of the BRB and the eye itself [92]. An additional advantage of the vitreous humor is its low protein content and the absence of blood vessels, which prevents the passage of blood-borne pathogenic microorganisms.

Currently, the vitreous humor, as a biological matrix, is primarily used not only for the analysis of administered drugs but also for the estimation of the PMI through the assessment of potassium levels in the vitreous humor.

The limitations of the vitreous humor include the relatively small volume of obtainable sample, which may be further affected by postmortem dehydration processes. Considering the cause and circumstances of death (e.g., sudden death or rupture of the eyeball), complete loss of the analytical material should be taken into account [93].

As an alternative matrix, the vitreous humor represents a highly valuable material in forensic investigations due to its favorable analytical properties; however, its applicability in toxicological analysis requires further research.

### 2.10. Human Breast Milk

Human breast milk is an optimal and irreplaceable source of nutrition for newborns, providing essential nutrients and containing a high concentration of lipoproteins. The World Health Organization (WHO) recommends exclusive breastfeeding during the first 6 months of life, followed by continued breastfeeding as a complementary source of nutrition alongside solid foods up to 2 years of age. According to current scientific knowledge, human breast milk is not considered a sterile fluid. In addition to nutritional components, it contains microorganisms, including commensal, probiotic, and pathogenic species.

As a complex biological fluid, human breast milk also contains components that support and modulate the immune and metabolic systems [95,96,97,98].

During lactation, numerous exogenous compounds may be transferred into breast milk following maternal exposure through unhealthy lifestyle behaviors, dietary habits, substance dependence, or environmental contamination, including exposure to areas affected by heavy metal pollution. Breastfed infants may be exposed to various substances, including pharmaceuticals, heavy metals, pesticides, nicotine, alcohol, and illicit drugs. Human breast milk is utilized as an alternative matrix in forensic toxicology for the detection of drugs with a short detection window. This analysis is useful for assessing recent maternal use of drugs or other substances within a short time frame (hours). Human breast milk, as an alternative matrix, provides information on infant exposure to potentially harmful substances, which may contribute to developmental disturbances affecting cognitive, motor, and intellectual functions [99,100]. The transfer of substances into human breast milk is determined by their physicochemical properties, including the degree of ionization (pKa), milk pH (6.5–6.8), molecular weight, and lipophilicity.

In recent years, a significant increase in the use of cannabis and cannabinoids has been observed, including among women of reproductive age.

Due to the high lipid content of human breast milk and the lipophilic nature of cannabinoids, the analysis of breast milk for the presence of cannabis and its derivatives is relatively straightforward.

The detection of cannabinoids in human breast milk is performed using analytical techniques such as ultra-performance liquid chromatography coupled with tandem mass spectrometry (UPLC–MS/MS) [101].

### 2.11. Meconium, Placenta and Umbilical Cord

Another example of the application of alternative biological materials in forensic and clinical toxicology is the analysis of meconium, placenta, and umbilical cord tissue.

Meconium, the first fecal material produced by a newborn, represents an excellent reservoir for drugs to which the fetus was exposed during the prenatal period [102]. The results obtained from meconium analysis, representing the accumulation of fecal material produced during pregnancy, enable the assessment of prenatal drug exposure over an extended time frame [103,104].

Neonatal urine is also used for the assessment of fetal exposure; however, the obtained results reflect exposure only during the previous 3–5 days. In contrast, meconium analysis provides information regarding potential drug exposure from the end of the second to the third trimester of pregnancy, offering a substantially longer detection window.

Studies have demonstrated that meconium exhibits higher sensitivity compared with umbilical cord tissue. In both matrices, the main limitation is the relatively short period and poorly understood mechanism of drug incorporation and deposition [104,105].

During pregnancy, tobacco exposure is associated with numerous fetal and obstetric complications. For the determination of, among others, nicotine and its metabolites (cotinine and hydroxycotinine) in placental and umbilical cord tissues, a method based on LC–MS/MS has been developed and validated [106]. Additionally, the aforementioned LC–MS/MS method has also been successfully applied for the quantitative determination of substances such as morphine, codeine, cocaine, benzoylecgonine, amphetamine, methamphetamine, methadone, and MDMA [107]. Unfortunately, despite the severe consequences for the fetus, illicit drug use during pregnancy remains insufficiently controlled. Potential adverse outcomes include fetal growth restriction, neonatal abstinence syndrome, impaired brain development, preterm birth, an increased risk of miscarriage, and even stillbirth [108].

### 2.12. Oral Fluid

In forensic science, the identification and analysis of biological fluids, including oral fluid, may play a crucial role in the resolution of forensic cases.

As a biological secretion, oral fluid is produced by three pairs of major salivary glands, namely the sublingual, parotid, and submandibular glands, along with numerous smaller submucosal salivary glands responsible for its production [109].

Due to the extensive vascularization of the salivary glands, oral fluid is referred to as an “ultrafiltrate” of blood, providing valuable information through the biomarkers it contains. These biomarkers reflect various pathological conditions of the body, including viral, bacterial, oncological, and cardiovascular diseases [110]. Drugs of abuse and pharmaceuticals are incorporated into oral fluid through passive diffusion, particularly for hydrophobic compounds, and through ultrafiltration of low-molecular-weight hydrophilic compounds [99,110]. Protein-bound drugs do not readily penetrate salivary tissues; therefore, only the unbound, non-ionized fraction of the drug can freely diffuse into oral fluid. Due to the relatively low pH of oral fluid (6.2–7.4), these substances may undergo ion trapping, resulting in falsely elevated concentrations compared with the actual drug concentration in blood.

This phenomenon is particularly relevant for weakly basic drugs of abuse that undergo ionization, including amphetamine and cocaine [111].

Saliva analysis is utilized, among other applications, in forensic investigations involving suspected abuse of anabolic–androgenic steroids. For this purpose, a sensitive and accurate method based on electrospray ionization liquid chromatography–tandem mass spectrometry (ESI-LC–MS/MS) was developed and validated for the determination of testosterone in serum and saliva. This method proved to be suitable for testosterone quantification across a broad concentration range, particularly in forensic medicine applications [112].

Saliva analysis is also performed in cases involving suspected overdose with certain drugs, including hypnotic agents such as zaleplon, zopiclone, and zolpidem. Postmortem analysis of oral fluid samples is conducted using liquid chromatography coupled with mass spectrometry techniques [113].

Oral fluid represents a non-invasive biological matrix that is easy to collect and does not require prior specialized training for sample acquisition [114].

### 2.13. Sweat

Sweat, as a biological fluid, is produced by apocrine and eccrine sweat glands. To maintain a constant body temperature, these glands are distributed throughout the body [99,115].

Sweat is utilized, among other applications, as an alternative matrix for drug monitoring and testing.

Sweat samples are collected using adhesive patches or sweat collection pads from body areas such as the back, chest, and arms. Although the exact mechanism of drug transfer into sweat remains unclear, passive diffusion is considered the primary pathway responsible for transport from the bloodstream into sweat glands. Drugs in their non-ionized form can penetrate into sweat and subsequently undergo ionization due to the pH conditions. Sweat exhibits low tonicity and a more acidic pH (mean pH = 6.3) compared with blood (mean pH = 7.4), allowing weakly basic drugs to readily diffuse into sweat as a result of the concentration gradient.

Another proposed mechanism of drug transfer involves diffusion through the stratum corneum of the epidermis to the skin surface, where the drug dissolves in sweat (transdermal migration).

Drug concentrations in sweat may be influenced by various factors, including age, sex, hydration status, ambient temperature, concomitant medication use, and diet. All of these factors may alter sweat pH values, which can range from 5.2 to 6.9 [99,115,116]. One of the major advantages of sweat as an alternative matrix is the non-invasive nature of sample collection, while the analytical process itself is safe and straightforward. Additionally, sweat samples have a relatively simple composition, which facilitates their analysis and minimizes the potential risk of contamination with pathogenic microorganisms [99]. A particularly important advantage of this matrix is its extended detection window compared with blood. The presence of a specific substance can be assessed even up to two weeks after exposure [117].

### 2.14. Evidence Materials

Biological evidence collected at crime scenes may have significant, and in some cases crucial, importance in the reconstruction of events during criminal proceedings. The key questions to be addressed include how the event occurred, the location of the incident, what happened, and who was responsible.

To achieve these objectives, forensic investigators utilize traces left at the scene, including dried stains of biological fluids such as saliva, blood, semen, and urine [118].

Materials collected from crime scenes are diverse, complex, and often of unknown origin. Analytical methods must therefore be carefully selected to enable accurate interpretation, evaluation, and analysis of evidence in a manner that most closely reflects the actual sequence of events. A key innovation in forensic science, particularly in the field of biological fluid analysis, is hyperspectral imaging (HSI). This advanced non-contact technology records both spectral and spatial information across a broad range of wavelengths, providing analysts with comprehensive data regarding the composition, characteristics, and distribution of biological fluids recovered from crime scenes. HSI encompasses the analysis of various biological fluids, including saliva, blood, urine, semen, vaginal secretions, and menstrual blood.

Compared with other more traditional analytical methods, HSI offers several advantages, including high sensitivity, enabling the detection of highly diluted or partially removed blood traces, the ability to differentiate between biological fluids even within complex mixtures, and a non-destructive approach to sample analysis. These features preserve sample integrity for potential further examinations during forensic investigations [119,120,121,122].

The presence of hematophagous insects at a crime scene may have potential significance for the detection of drugs of abuse. However, to date, no studies have been conducted to evaluate the usefulness of insect-derived trace evidence for forensic toxicological investigations. However, the results of an experimental laboratory study involving the blowfly species *Calliphora vomitoria* indicate its potential future application in real forensic cases where traditional biological matrices are unavailable [123]. The analysis of evidentiary materials collected at crime scenes has several limitations and disadvantages. The most common factors affecting analytical results include environmental conditions, substrate characteristics, aging processes of biological fluids, the presence of animal-derived biological fluids, and non-biological fluids, all of which may interfere with the analysis and lead to inaccurate results [124].

In cases where analytical results raise uncertainties, the only available solution may involve the exhumation of an embalmed body or the analysis of samples previously preserved in formalin. When interpreting post-embalming analyses, the interaction between drugs and formaldehyde must be considered, as formaldehyde is a highly reactive chemical compound. Therefore, the formalin solution must be thoroughly examined for chemical derivatives and potential degradation products to ensure accurate interpretation of analytical results [125,126]. Studies concerning chlorpromazine, promethazine, and levomepromazine demonstrated a reduction in analyte concentrations compared with sample concentrations before formaldehyde fixation, with decreases reaching up to 39%. All investigated substances exhibited reduced stability following formaldehyde exposure, significantly affecting the analytical results [127].

In summary, based on the above review of alternative matrices, Table 3 presents a compilation of the most frequently used matrices for the determination of toxic substances, including their respective advantages and limitations, while Table 4 details the analytical methods and their implications.

## 3. Genetic Changes Resulting from Poisoning or Substance Abuse

The advancement of forensic sciences is associated with the continuous discovery and development of new techniques aimed at assisting in the resolution of the most challenging criminal cases. One such method is the application of microRNA (miRNA) analysis [138]. MiRNAs are small, non-coding RNA (ncRNA) molecules approximately 22 nucleotides in length that exhibit greater resistance to degradation compared with messenger RNA (mRNA) [139]. The increased stability of miRNA is particularly important in cases where biological material may be exposed to adverse environmental conditions. Due to its diverse biological functions, including the regulation of cellular processes such as cell differentiation, proliferation, and apoptosis, miRNA has found applications in forensic science as a marker for the identification of biological fluid sources. Furthermore, it can be used to distinguish monozygotic twins and may also serve as a tool for estimating the postmortem interval and determining the cause of death [140,141,142].

Determining the tissue origin of individual biological fluids recovered from a crime scene is highly valuable for event reconstruction [143]. For most biological fluids, including blood, urine, semen, and saliva, commercially available immunochromatographic tests are used for identification based on protein antigens. However, these methods have several limitations, including cross-reactivity with other biological fluids and the occurrence of false-positive results. The most significant disadvantage of these methods is the degradation of the analyzed biological sample, which may pose a major challenge during subsequent stages of analysis when only a limited amount of material is available [144,145]. An important issue in forensic investigations is the differentiation of monozygotic (MZ) twins. MZs twins are genetically identical; therefore, traditional DNA profiling methods are insufficient for their differentiation. For this purpose, selected miRNAs were investigated, including miR-151a-3p, miR-3653-3p, miR-142-3p, miR-4325, miR-16-5p, let-7i-5p, miR-222-3p, miR-550b-3p, miR-4791, and miR-27a-3p. The results of the conducted studies demonstrated differences in miRNA expression patterns among the examined twin pairs. These findings were confirmed using quantitative real-time PCR (qRT-PCR), indicating the potential application of miRNA analysis for forensic identification of MZs twins [146]. Other studies have demonstrated that opioid drugs also influence miRNAs (sequences consisting of 17–22 nucleotides), which function as pleiotropic regulators of mRNA through imperfect base pairing, predominantly within the 3′ untranslated regions (3′UTRs) of target genes. miRNAs are key modulators of intercellular communication in the brain. Exposure to opioid drugs markedly alters miRNA expression across multiple brain regions, with the most pronounced changes observed in the prefrontal cortex, as well as in the striatum and nucleus accumbens [147]. Furthermore, post-mortem studies involving peripheral blood samples have identified brain-specific miRNAs that may potentially serve as biomarkers for the assessment of traumatic brain injury. Among the markers demonstrating the greatest diagnostic potential, six top-ranked miRNAs were identified: miR-124-3p, miR-219a-5p, miR-9-5p, miR-9-3p, miR-137, and miR-128-3p. Additionally, these miRNAs may function as molecular mediators of physiological alterations that occur following traumatic injury [148].

Deoxyribonucleic acid (DNA) methylation, histone modifications (including acetylation, methylation, and phosphorylation), and chromatin remodeling are all processes encompassed within the broad field of epigenetics. Epigenetics focuses on the study of mechanisms that influence variable patterns of gene expression without altering the underlying DNA sequence itself [149,150].

In forensic epigenetics, applications include, among others, the estimation of the age of an unknown individual who has left biological traces at a crime scene; identification of body fluids and tissues; determination of the PMI; and age assessment. Epigenetic processes may be either inherited or acquired throughout an individual’s lifetime [149,151]. Environmental factors, stress, drugs, medications, diet, lifestyle, and behaviour all contribute to epigenetic alterations. Continuously transmitted signals induce specific epigenetic modifications that enable adaptation to a given environment by altering gene expression patterns without changing the underlying DNA sequence [149].

Exposure to illicit substances induces alterations in gene expression through epigenetic modifications occurring in various brain regions, resulting in changes in neuronal function, structure, and neural circuitry. These epigenetic changes are estimated to persist for months or even years [152].

One example of epigenetic alterations is chronic, repeated cocaine use, which affects the expression of genes involved in neuronal adaptations. Cocaine use disorder (CUD) is associated with a loss of control over cocaine intake and is linked to neurobiological changes occurring in the brain, including structural, functional, and molecular alterations. Chronic cocaine use increases miR-212 expression in the dorsal striatum, leading to a reduction in motivation [153]. Studies investigating cocaine abuse have demonstrated its effects on DNA methylation and the expression levels of DNA methyltransferases (Dnmt1, Dnmt3a) and ten–eleven translocation (Tet) demethylases, thereby directly altering DNA methylation (DNAm) homeostasis. It has been shown that DNAm patterns are tissue-specific, and the estimation of differential methylation signatures associated with CUD in brain tissue based solely on blood samples may not be sufficient. Therefore, profiling DNAm levels directly in brain tissue is of significant importance. Available data indicate that epigenomic studies in CUD have been conducted using post-mortem brain tissue samples obtained from the caudate nucleus and nucleus accumbens. A breakthrough finding was the identification of a hypermethylated region within the tyrosine hydroxylase gene. In epigenome-wide analyses of CUD-associated alterations, this region demonstrated a greater prevalence of hypermethylation than hypomethylation at the level of differentially methylated regions (DMRs) in BA9 (DMR—differentially methylated regions; BA9—Brodmann area 9). In subsequent studies, epigenetic clocks were used to assess biological age, revealing a tendency toward accelerated epigenetic aging in individuals with CUD within the BA9 region [154,155,156]. Increasingly, epigenetic modifications associated with alterations in mood states are being observed. Post-mortem studies have provided evidence confirming alterations in gene expression within brain tissue and peripheral cells in patients with mood disorders [157]. Further epigenetic changes have been observed in individuals with suicidal tendencies, including alterations in genes such as GRIK2, BEGAIN, BDNF, and TrkB, which are involved in neuronal survival and viability. The observed epigenetic alterations involved miRNA methylation processes and histone modifications. Following analyses of the prefrontal cortex in individuals who died by suicide, researchers demonstrated reduced DNA methylation levels, indicating the presence of region-specific epigenetic changes within the brain in these individuals [158].

Conversely, other studies conducted in the ventral prefrontal cortex of individuals with depression who died by suicide revealed increased DNA methylation levels [159]. Another important aspect is alcohol use disorder (AUD), which is defined as a chronic, relapsing brain disorder. Studies have demonstrated that AUD is closely associated with alterations in DNA methylation processes. DNA methylation plays a role in the regulation of neurosteroid biosynthesis and GABAergic neurotransmission; however, the underlying mechanisms remain incompletely understood. The findings indicate a reduction in the expression of neurosteroidogenic enzymes within the cerebellum of individuals with AUD, which may be associated with the stimulation of neurosteroidogenesis resulting from chronic and acute alcohol exposure [160].The response observed following long-term alcohol consumption is presumably associated with the mechanisms underlying addiction, providing a potential rationale for the use of synthetic neurosteroids (i.e., allopregnanolone) in the pharmacotherapy of AUD [161]. An analysis of epigenetic mechanisms involving key enzymes of neurosteroidogenesis revealed increased DNA methylation levels within the region of the gene encoding 3α-HSD in the cerebellum of individuals with AUD [162]. Beyond AUD, epigenetic mechanisms are increasingly being linked to other molecular pathways, including those involved in hepatic fibrosis and liver injury resulting from chronic alcohol consumption, such as alcoholic hepatitis (AH—human alcoholic hepatitis) [163]. Gene expression studies conducted in patients with AH have identified receptors belonging to the tumour necrosis factor (TNF) family. Among the TNF superfamily receptors, Fn14 was the only receptor found to be upregulated in individuals with AH, in contrast to other liver diseases. Fn14 protein expression was localized within fibrogenic regions and clusters of hepatocytes [164].

Interest in miRNAs and the application of epigenetics in forensic sciences has increased significantly in recent years. The rapid development of this field suggests that current limitations related to post-mortem stability and tissue specificity may be overcome in the future. However, at present, the obtained results should be interpreted and applied cautiously in forensic investigations [165].

## 4. Conclusions

In forensic toxicology, analyses may involve a wide variety of biological materials collected both antemortem and postmortem. Following death, numerous biochemical and biological processes occur that may significantly influence the post-mortem concentration of toxic substances. These processes may render the quantitative determination of individual drugs unreliable and, in some cases, may result in drugs becoming undetectable, even when multiple analytical methods are applied, regardless of the biological matrix examined. Problems may arise, among others, from alterations in drug concentrations resulting from bacterial degradation, residual enzymatic activity within tissues, or post-mortem redistribution (the movement of a drug along a concentration gradient from tissues with higher concentrations to those with lower concentrations). In many analytical techniques, interference may occur due to co-extracted putrefactive compounds, which can mask or alter the detection of the drug [166].

As decomposition of the body progresses, the number of factors that must be considered for the appropriate selection of samples for toxicological analysis increases. Addressing the challenges encountered often requires knowledge not only of toxicology but also of numerous other disciplines, including biology, chemistry, biochemistry, microbiology, entomology, forensic medicine, and taphonomy. In situations where the availability of traditional and alternative methods of human identification is severely limited, methods from the field of forensic anthropology are employed. The Board of the Forensic Anthropology Society of Europe (FASE), in its position statement, argues that forensic anthropological methods can be valuable for physicians and law enforcement agencies by providing information sufficient for identification purposes, particularly in cases involving mass disasters or mass fatalities resulting from criminal acts [167].

The Organization of Scientific Area Committees for Forensic Science (OSAC) recommends further research into alternative matrices to facilitate future advancements in forensic toxicology. OSAC documents and publicly provides the forensic science community with research and development (R&D—Research and Development) needs, including those related to alternative matrices [Organization of Scientific Area Committees for Forensic Sciences (OSAC) (2021), *OSAC Research and Development Needs—Alternative Matrices*] [168].

## Figures and Tables

**Table 1:** Types and characteristics of post-mortem redistribution (PMR) mechanisms.

No.	Type of PMR Mechanism	Characteristics of the PMR Mechanism	References
1.	Hypostasis and blood coagulation	Blood clot formation results in a non-uniform distribution of the drug concentration. If a blood clot is detected during sample collection, the measured concentration of the analyte may be inaccurate due to its uneven distribution between the blood serum and erythrocytes. A substantial proportion of erythrocytes becomes entrapped within the clot, while the extent of their release influences the composition of the liquid phase of the specimen.	[11,13]
2.	Passive diffusion	Postmortem blood flow from highly perfused reservoir organs, such as the heart, lungs, liver, and gastrointestinal tract, may lead to increased concentrations of the analyte in central blood. Consequently, peripheral blood, being anatomically separated from the thoracic cavity, is considered a more reliable indicator of the antemortem blood concentration of the substance.	[11,13,14]
3.	Physical redistribution	This mechanism occurs during the first few hours after death, before the onset of putrefaction.	[11,13,14]
4.	Gravity	Diffusion of drugs from the lungs into the left ventricle via the pulmonary venous circulation may occur, particularly in bodies in the supine position. Drugs commonly used in clinical practice, including local anesthetics, tricyclic antidepressants, opioids, and cardiac glycosides, have a propensity to accumulate in the myocardium. Following death, their redistribution into cardiac blood may result in falsely elevated concentrations of the analyte, potentially leading to overestimation of the measured drug concentration.	[11,13,14]
5.	Bacterial invasion	This process begins immediately after death. Microorganisms are capable of metabolizing a wide range of drugs. Particular attention should be paid to drugs whose chemical structure renders them more susceptible to bacterial metabolism. These include certain antipsychotic agents containing sulfur moieties and nitrobenzodiazepines containing nitrogen, which may undergo postmortem bacterial biotransformation.	[11,12,13,14]
6.	Bacterial metabolism	It may contribute to an increase in ethanol concentration, a phenomenon of particular importance in the forensic investigation of road traffic fatalities and other traffic-related incidents.	[12,13,14]
7.	Cellular autolysis	It results in a rapid increase in drug concentrations in both central and peripheral blood.	[11,13]
8.	Agonal aspiration	Redistribution of drugs from the adjacent lungs may lead to falsely elevated drug concentrations in blood.	[11,15,16]
9.	Additional factors: Chemical and pharmacokinetic properties of the drug	Physicochemical and pharmacokinetic properties, including acidity, alkalinity, pKa, lipophilicity, protein binding, volume of distribution (Vd), and residual metabolic activity, influence the extent of PMR. PMR predominantly affects drugs that are lipophilic, basic, highly protein-bound, and characterized by a large volume of distribution (Vd). Lipophilic weak bases preferentially accumulate in solid organs, particularly the liver, lungs, and heart, thereby creating concentration gradients that promote passive diffusion after death. As the postmortem interval increases, intracellular pH decreases rapidly, resulting in increased ionization of basic drugs. Following complete cellular breakdown, these compounds are readily released and redistributed, potentially producing falsely elevated concentrations of the analyte in postmortem blood. Drugs with a volume of distribution (Vd) greater than 3 L/kg are extensively distributed into tissues and exhibit the greatest potential for PMR. Their release from tissue reservoirs into the plasma after death contributes to increased postmortem blood concentrations of the analyte.	[11,12,15,16]

**Table 2:** Routes of drug incorporation into nails.

No.	Type of Drug Incorporation Pathway	Mechanism of Drug Incorporation	References
1.	Transungual absorption pathway	The active pharmaceutical ingredient is incorporated into the developing nail plate during nail growth. As keratinization progresses, it is transported from the nail matrix and lunula toward the distal free edge of the nail.	[25,31,34]
2.	Incorporation into the developing nail plate from the germinal matrix.	The active substance reaches the nail matrix through systemic circulation and is subsequently incorporated into the keratin of the developing nail plate during its formation.	[37,38,39]
3.	Contamination of the nail plate via sweat exposure	The presence of the substance on the nail plate surface is attributable to deposition via sweat secretions. This mechanism does not constitute genuine incorporation of the drug into the nail matrix or plate structure and is regarded as a form of exogenous contamination.	[37]

**Table 3:** Compilation of the most frequently used matrices for the determination of toxic substances, including their respective advantages and limitations.

Type of Matrix	Disadvantages	References	Advantages	References
HAIR	- External contamination may lead to false-positive results. Without prior decontamination procedures, the obtained results can only be used for qualitative analysis. Melanin content (hair color) → the stronger the pigmentation of the hair, the greater the amount of drug bound compared with hair exhibiting lower pigmentation. - The mechanism of drug incorporation remains unclear. - Hair growth rate influences variability in drug concentrations. - Hair washing procedures, thermal cosmetic treatments, and the use of bleaching products containing strong alkaline agents significantly affect the amount and stability of drugs present in the hair matrix. - Exposure to sunlight, weather conditions, and environmental contaminants may substantially damage the hair cuticle, contributing to drug photodegradation.	[32,86,99,128,129,130,131]	- The possibility of detecting substances in their parent form or as metabolites. - Toxicological analysis of the hair root is used for the detection of acute postmortem intoxication, whereas analysis of the hair shaft can provide evidence of long-term drug use. - Enables the assessment of previous drug exposure with a long detection window (weeks or months, depending on hair length). - Collected samples remain stable during storage at room temperature and can be stored in sealed envelopes. - Allows easy transportation due to its durable structure. - Represents a matrix resistant to decomposition. - Due to the acidic and hydrophobic properties of melanin, hair exhibits greater affinity for alkaline substances, resulting in improved detection of compounds such as cocaine, ketamine, and codeine. - Allows easy and non-invasive sample collection without the need for specialized training. - Associated with a minimal risk of infection.	[31,32,33,86,99,129,132,133,134]
NAILS	- External contamination may result in false-positive findings. - Concentrations of analyzed substances may differ between fingernails and toenails. - The mechanism of drug incorporation into the nail remains unclear. - Significant physiological, biochemical, and pharmacokinetic differences may contribute to inaccurate quantitative drug analysis. - Age and metabolic disorders may result in elevated concentrations of the analyzed substance. - Sex-related differences may influence the absorption, distribution, metabolism, and excretion of drugs.	[41,86,132]	- Collected samples remain stable during storage at room temperature. - Toenail samples can document exposure to a specific substance for up to 14 months. - Retrospective monitoring using fingernail clippings allows assessment of exposure over a period of approximately 5 months. - Continuous accumulation of substances over an extended period of time.	[37,38,39,40,86]
BONES	- Ability of microorganisms to colonize and degrade bone tissue. - Tissue susceptible to chemical, physical, and biological factors, which may alter its original composition.	[44]	- Enables the assessment of previous drug exposure, even after a prolonged PMI, including several years after death. - Exhibits high resistance to putrefactive processes. - Tissue is well protected within the body against exogenous factors, including external contaminants.	[46,47,48]
BONE MARROW	- PMR (potential misinterpretation of postmortem drug concentrations). - Tissue degradation (formation of interfering degradation products). - Evaporation. - Neoformation. - Limited pharmacokinetic data regarding bone marrow. - Cellular autolysis (residual enzymatic activity during the early postmortem period may lead to drug biotransformation). - Microbial invasion. - Putrefactive processes. - Short period of suitability for toxicological analysis. - Not routinely examined during autopsy procedures. - Environmental conditions, including temperature, humidity level, and burial conditions, may interfere with the performed analysis.	[57,61,135]	- Exhibits a high degree of vascularization and contains a lipid matrix that functions as a drug reservoir. - The surrounding bone provides a protective barrier against exogenous contamination, shielding the tissue from postmortem effects of bacteria, fungi, plants, and animals. - Histopathological analysis provides information regarding the PMI and previous pathological conditions. - Easily collected for analytical purposes. - Demonstrates good resistance to autolysis and contamination.	[56,57,135,136]
CARTILAGE TISSUE	- Studies utilizing cartilage tissue as a matrix in forensic medicine are still ongoing and continue to be refined. ⟶ Insufficient research has been conducted to adequately evaluate the usefulness of this matrix.		- Enables the assessment of previous toxic exposure to substances. - Simple sample preparation procedure before analysis. - Analysis duration. - Potential matrix for the determination of the PMI. - Cartilage tissue may be used for age estimation.	[63,68,69]
TEETH	- The formation of dental plaque may act as a barrier for drugs and illicit substances, limiting their incorporation into the tooth enamel. - When interpreting results, the routes and rates of absorption of different drugs, as well as physicochemical differences between the tooth root, tooth crown, and carious material, should be taken into consideration. - Tooth tissue may be unavailable due to the absence of dentition or as a result of environmental factors.	[70,74]	- Enables the assessment of previous drug exposure (hard dental tissue) and recent drug use (dental biofilm). - The simultaneous use of hard dental tissue and dental biofilm matrices provides an extended detection window. - Dental biofilm may act as a drug reservoir, facilitating drug transport into the tooth enamel. - Application of methods such as the PAR and AAR for age estimation of the deceased at the time of death.	[70,78,79]
FINGERPRINTS	- Presence of sweat traces from sebaceous and apocrine glands on the surface of the hands due to contact with other body areas. - Numerous factors may alter the original composition of sweat, including environmental conditions, contamination, the type of surface on which the print is deposited, airflow, and weather-related factors such as UV radiation, humidity, and temperature. - Instability resulting from changes occurring between the fingerprint residue and the contact surface.	[80,81]	- Enables the assessment of previous drug exposure. - Detection of drugs from fingerprint samples. - Analysis of metabolites of previously consumed substances.	[81,82]
CEREBROSPINAL FLUID (CSF)	Due to the complexity of procedures involved in the pre-analytical assessment preceding postmortem cerebrospinal fluid analysis, as well as the lack of sufficiently advanced analytical techniques, it remains difficult to determine the applicability of cerebrospinal fluid as an alternative matrix for toxicological investigations, including the detection of narcotic and psychoactive substances.	[87,90]	Deep anatomical location within the spinal canal protects against external contamination.	[88]
VITREOUS HUMOR	- Small sample volume available for collection, which is additionally affected by the postmortem dehydration process. - Depending on the cause and circumstances of death (e.g., violent death or rupture of the eyeball), complete loss of the analytical material may occur.	[93]	- Enables the detection of drugs, illicit substances, and volatile compounds. - Resistant to postmortem processes involving the autosynthesis of endogenous compounds. - Exhibits extended stability, allowing a longer period for analytical examination. - Its anatomical location provides effective protection against putrefaction, external contamination, and microbial activity. - Lacks many enzymes, making it an excellent matrix for the analysis of substances with rapid metabolism (e.g., cocaine). - An additional advantage is its low protein content and the absence of blood vessels, which prevents the penetration of blood-borne pathogenic microorganisms. - Low protein content positively influences qualitative drug analysis, as protein binding is limited or absent. - Can be used for PMI estimation.	[91,92,93,94]
HUMAN BREAST MILK	- Short detection window, making it useful primarily for the assessment of recent maternal drug use (within hours). - The transport of substances into breast milk is not fully understood and is influenced by various physicochemical properties, including the degree of ionization (pKa), milk pH (6.5–6.8), molecular weight, and lipophilicity.	[99,101]	- Non-invasive and easy sample collection. Enables the assessment of infant exposure to harmful substances.	[99]
MECONIUM UMBILICAL CORD TISSUE PLACENTA	- Meconium may be expelled into the uterus, resulting in the potential loss of the entire sample material. - The timing and mechanism of drug deposition are not yet fully understood. - Umbilical cord tissue analysis enables drug detection only for exposure occurring during the third trimester of pregnancy.	[104]	- Enables the assessment of long-term drug exposure. - Provides a wide detection window, reflecting drug exposure occurring from the end of the second trimester through the third trimester of pregnancy. - In analytical studies, meconium demonstrates significantly higher sensitivity compared with umbilical cord tissue.	[102,104]
ORAL FLUID	- Obtained results may be affected by confounding factors, including drug pKa, sample pH, protein binding, and the method of sample collection. - The phenomenon of ion trapping may result in falsely elevated drug concentrations compared with the actual blood concentrations (particularly in the case of amphetamine and cocaine).	[111,137]	A simple, rapid, readily accessible, and non-invasive biological matrix.	[137]
SWEAT	- The mechanism of drug excretion into sweat remains not fully understood. - Factors limiting transport mechanisms (passive diffusion/transdermal migration), including pKa, molecular weight, protein binding, and lipophilicity. - Low analyte concentrations, requiring the application of more sensitive analytical methods. - Internal and external factors affecting sample quantity. - Drug concentrations in sweat may be influenced by factors such as age, sex, hydration status, ambient temperature, concomitant medication use, and diet. All of these factors may alter pH values ranging from 5.2 to 6.9, potentially interfering with analytical results.	[99,115,116,117]	- Extended detection window of approximately two weeks. - Minimal risk of potential contamination with pathogenic microorganisms. - Sweat samples have a relatively simple composition. - Non-invasive and straightforward sample collection, with the analytical procedure itself being safe and simple to perform. - Easy sample storage. - Facilitates the acquisition of a comprehensive record of drug exposure.	[99,117]
EVIDENCE MATERIALS	- Diversity and complexity of analyzed materials with unknown characteristics. - The need for precise selection of analytical methods to ensure interpretation, evaluation, and analysis that most accurately reflect the probable sequence of events. - Influence of environmental conditions. - Substrate characteristics. - Aging processes of biological fluids. - Presence of animal-derived biological fluids and non-biological fluids, which may interfere with analysis and lead to inaccurate results.	[119,120,121,122,124]	Enables the reconstruction of events during criminal proceedings, including the determination of the cause, location, and perpetrator.	[118]

**Table 4:** Summary of methods used and their implications for the matrices employed in the determination of toxic substances.

Matrix Type	Method Type	Advantages	Limitations	References
Hair	Segmental hair analysis	Enables chronological reconstruction of exposure to psychoactive substances through the analysis of consecutive hair segments. It allows the identification of both chronic substance use and single exposure events. Furthermore, it enhances the evidential value of hair specimens in forensic investigations.	The accuracy of interpretation depends on the individual hair growth rate and the proportion of hairs in the anagen, catagen, and telogen phases. The method requires an adequately long hair sample and meticulous preparation of the analytical specimen.	[31,32,33]
	High-performance liquid chromatography (HPLC)	It is characterized by high reproducibility and the ability to determine thermolabile and non-volatile compounds. It is widely used for the analysis of pharmaceuticals and psychoactive substances in biological matrices.	In its conventional form, it exhibits lower identification specificity compared with techniques coupled with mass spectrometry. It often requires additional confirmation of the obtained results using methods with higher selectivity.	[25,26,27,28,29,30]
	Gas chromatography–mass spectrometry (GC–MS)	Considered one of the reference methods in forensic toxicology, GC–MS provides high sensitivity, analytical specificity, and reliable identification of a wide range of psychoactive substances and their metabolites. It enables the analysis of complex mixtures of chemical compounds with high accuracy and selectivity.	A major limitation of this technique is the requirement for analytes to be volatile or amenable to derivatization prior to analysis. The sample preparation procedure is often time-consuming and requires specialized instrumentation as well as highly trained personnel.	[25,27,28,30]
	High-performance liquid chromatography coupled with tandem mass spectrometry (HPLC–MS/MS, LC–MS/MS)	It is characterized by exceptionally high sensitivity, selectivity, and low limits of detection. The technique enables the simultaneous determination of multiple analytes, including substances present at trace concentrations, without the need for derivatization. It is considered one of the most valuable analytical approaches in contemporary analytical toxicology.	The high costs associated with instrumentation, maintenance, and method validation limit its widespread availability. The analysis requires specialized software and highly qualified personnel with expertise in the field.	[25,26,27,28,29]
	Gas chromatography coupled with tandem mass spectrometry (GC–MS/MS).	It provides exceptionally high sensitivity and selectivity of analyte determination while effectively minimizing the impact of matrix interferences. The technique is particularly valuable for the analysis of trace levels of psychoactive substances and for the confirmation of screening test results.	Similar to GC–MS, this technique requires the analysis of volatile compounds or their prior derivatization. The analytical procedure is costly, time-consuming, and requires advanced laboratory infrastructure and specialized analytical facilities.	[25,26,27,28,29]
Nails	Nails as a keratinized biological specimen matrix	Nails represent a stable biological matrix enabling retrospective assessment of exposure to pharmaceuticals and toxic substances. They allow the detection of analytes over extended periods, reaching approximately 5 months in fingernails and up to 14 months in toenails. Due to their high resistance to autolytic processes and post-mortem degradation, nails are particularly valuable in forensic toxicology. Sample collection is non-invasive, and nail specimens exhibit high stability during transportation and long-term storage. Nail analysis represents a valuable complementary approach to hair testing, particularly in cases where hair samples are unavailable or insufficient for analytical purposes.	The mechanism of drug incorporation into the nail plate has not yet been fully elucidated, which limits the ability to perform an unambiguous quantitative interpretation of the obtained results. Analyte concentrations are influenced by numerous physiological, biochemical, and pharmacokinetic factors, including age, sex, nail growth rate, and hepatic and renal function disorders. Substance concentrations may differ between fingernails and toenails, requiring the sampling site to be taken into consideration during result interpretation. A linear relationship between the duration of drug use and its concentration in nails cannot be assumed, which limits the possibility of quantitatively assessing the extent of exposure. Compared with hair analysis, this approach is less extensively validated and is supported by a smaller body of reference data.	[37,38,39,40,41]
Bones	Gas chromatography–mass spectrometry (GC–MS) in bone analysis.	It is an established and validated technique used in forensic toxicology. It is characterized by high selectivity and enables the unequivocal identification of a wide range of psychoactive substances, including opioids, benzodiazepines, and antidepressants. The method allows for the analysis of complex mixtures of chemical compounds present in bone tissue and enhances the reliability of toxicological assessments.	The application of GC–MS is limited for non-volatile, thermolabile, or highly polar compounds. In many cases, derivatization of analytes is required, which increases the complexity of the sample preparation procedure and may extend the overall analysis time. The technique also requires specialized instrumentation and appropriately trained personnel.	[49,50,51,52]
	Liquid chromatography–tandem mass spectrometry (LC–MS/MS) in bone analysis.	It demonstrates exceptionally high sensitivity and selectivity, enabling the determination of compounds present at low concentrations in degraded biological material. The technique is particularly suitable for the analysis of polar, thermolabile, and non-volatile compounds, for which the application of GC–MS is challenging. It does not require prior derivatization of analytes, thereby simplifying sample preparation and reducing the risk of analyte loss during the analytical procedure.	The high cost of instrumentation and technical requirements limit the availability of this method in some toxicology laboratories. Interpretation of results may be challenging due to the lack of comprehensive standardization of procedures for all types of bone samples and variability associated with the degree of degradation of the biological material.	[53]
	Comparison of GC–MS and LC–MS/MS in the analysis of toxicological substances in bone tissue.	The application of both techniques enables the analysis of a broad range of chemical compounds with diverse physicochemical properties. The combination of the high selectivity of GC–MS with the enhanced sensitivity and versatility of LC–MS/MS expands the diagnostic capabilities of contemporary forensic toxicology.	The selection of an appropriate analytical technique requires consideration of the physicochemical properties of the target analyte, the type of biological matrix, and the extent of sample degradation. The absence of a single universal method applicable to all compounds limits the possibility of standardizing analytical procedures.	[54,55]
Bone marrow	Validated analytical methods for the extraction and determination of drugs in bone marrow samples	The development and validation of analytical methods enable the potential application of bone marrow as a biological matrix in routine forensic toxicology procedures. Evaluation of validation parameters, including sensitivity, accuracy, precision, linearity, and matrix effects, allows the assessment of method suitability for the determination of pharmaceuticals, including antidepressant and antipsychotic agents.	Methods for the analysis of bone marrow still require further investigation and extensive validation using human specimens. The limited number of reference studies hinders the establishment of threshold values and the clinical and forensic interpretation of results. Analytical procedures may require individual optimization depending on the type of analyte being determined and the extent of sample degradation.	[57,58,59,60,61,62]
Cartilage tissue	Determination of ethanol in cartilage tissue using GC–FID.	The application of gas chromatography with flame ionization detection (GC–FID) enables the detection of ethanol in an alternative biological matrix. Ethanol concentrations obtained from costal cartilage demonstrated a correlation with concentrations determined in femoral blood and urine, indicating the potential use of this tissue in the diagnosis of alcohol intoxication.	The method requires further investigation to determine the impact of post-mortem processes on ethanol stability in cartilage tissue. Due to the potential for ethanol formation and degradation during the post-mortem interval, careful interpretation of results is required, with consideration of relevant environmental factors.	[64,65]
	Ethanol determination using gas chromatography with flame ionization detection (GC–FID	Results obtained from costal cartilage demonstrate agreement with ethanol concentrations determined in femoral blood and urine. The GC–FID technique provides high sensitivity and analytical specificity.	The method requires specialized laboratory instrumentation and appropriately validated analytical procedures. The application of cartilage tissue as a biological matrix still requires further clinical and forensic investigations.	[64,65]
	Quantitative determination of sodium nitrite (NaNO_2_) in cartilage tissue	It enables the identification of fatal sodium nitrite intoxication cases, even when using an alternative biological matrix. The obtained results indicate its potential diagnostic applicability in forensic toxicology.	Current reports are based on individual case studies and require confirmation in investigations involving larger sample sets. Standardization of the analytical methodology remains lacking.	[66,67]
	Estimation of the post-mortem interval (PMI) using cartilage tissue	The biological properties of cartilage indicate its potential use as a stable matrix for post-mortem interval (PMI) estimation. The prolonged preservation of cellular viability enhances its applicability in determining the time since death.	The method has not yet been standardized for routine forensic medical practice. The reliability of determinations is influenced by environmental factors, particularly temperature and the post-mortem interval.	[68,69]
Teeth	Liquid chromatography coupled with tandem mass spectrometry (LC–MS/MS)	It provides high sensitivity and specificity for the determination of methadone, morphine, and their metabolites in dental plaque following acetonitrile extraction. The method enables the detection and analysis of trace amounts of psychoactive substances.	The method requires specialized instrumentation, appropriately validated analytical procedures, and extensive sample preparation, which limits its availability outside specialized laboratories.	[72,73]
	Gas chromatography coupled with mass spectrometry (GC–MS)	It enables the assessment of chronic exposure to psychoactive substances. Analytical results obtained from dental tissues demonstrate agreement with concentrations determined in body fluids and hair samples. The method exhibits high diagnostic value in retrospective analyses.	The method requires advanced laboratory infrastructure and specialized interpretation of results. It does not provide information regarding the timing of the last substance intake.	[75]
	Combined use of dental hard tissue and dental biofilm	It enables the simultaneous assessment of previous and recent exposure to psychoactive substances. By extending the detection window, it significantly enhances the evidential value of toxicological findings.	The incorporation of substances into dental enamel may be limited by the presence of dental deposits, which can affect the amount of deposited analyte.	[70,71]
	Assessment of post-mortem pink discoloration of teeth.	It may provide supplementary information regarding the circumstances of death, the post-mortem interval, and the potential mechanism of death, particularly in cases involving prolonged exposure of the remains to an aquatic environment.	Currently, there is insufficient conclusive scientific evidence confirming a direct association between pink discoloration of teeth and a specific cause of death; therefore, this finding should be regarded only as supportive evidence.	[76,77]
	Pulp/tooth area ratio (PAR) method for age estimation	A non-invasive age estimation method based on the ratio between pulp area and tooth area. It demonstrates high reliability and independence from the sex of the examined individual, which enhances its applicability in forensic identification.	The accuracy of the method depends on the ability to correctly assess the pulp cavity and the quality of the examined material.	[78]
	Aspartic acid racemization analysis (AAR—Aspartic Acid Racemization).	One of the most valuable chemical methods for age estimation. It is based on the predictable increase in the proportion of the D-isomer of aspartic acid with advancing age, providing high diagnostic value in the identification of deceased individuals.	The method requires specialized chemical analyses and appropriately prepared biological material, which limits its routine application.	[79]
Fingerprints as forensic evidence	Dactyloscopy (fingerprint identification).	It represents the gold standard in personal identification within forensic science. The method is based on the uniqueness and permanence of fingerprint characteristics, providing high evidential value during the comparison of latent prints with reference samples.	Its application is primarily limited to individual identification and does not provide information regarding exposure to psychoactive substances or the time at which the trace was deposited.	[80]
	Liquid chromatography–tandem mass spectrometry (LC–MS/MS).	It is characterized by high analytical sensitivity and specificity, enabling the detection of drugs even following a single intake. The method allows assessment of whether an individual who left a fingerprint may have been under the influence of psychoactive substances at the time of the incident.	The method requires specialized instrumentation and highly qualified personnel. Interpretation of results may be challenging due to variability in the chemical composition of fingerprint residues.	[81,82]
Cerebrospinal fluid (CSF)	Post-mortem imaging (PMMR, PMCT) as a complementary examination.	A non-invasive and reproducible method supporting conventional autopsy procedures. It enables permanent archiving of imaging data and facilitates the assessment of the cause and mechanism of death. It may serve as a valuable complement to the interpretation of toxicological findings.	It does not replace toxicological examinations or chemical analyses of cerebrospinal fluid. The method requires access to highly specialized diagnostic imaging equipment and appropriate interpretation of imaging findings.	[89]
	Determination of ethanol concentration in cerebrospinal fluid.	It demonstrates a very strong positive correlation with blood ethanol concentrations, confirming the diagnostic utility of cerebrospinal fluid (CSF) in cases of ethanol intoxication. It may serve as a reliable alternative biological specimen, particularly in cases involving advanced decomposition of the remains.	Current evidence primarily concerns ethanol determination, whereas the applicability of cerebrospinal fluid analysis for other psychoactive substances remains insufficiently investigated.	[88]
Vitreous humor	Analysis of drugs, illicit substances, and volatile compounds in vitreous humor.	It enables the determination of a broad spectrum of toxicologically relevant substances, including pharmaceuticals, illicit drugs, and volatile compounds. The limited enzymatic activity of vitreous humor promotes the preservation of rapidly metabolized compounds, thereby increasing the reliability of analytical results.	Substance concentrations depend on the pharmacokinetic and physicochemical properties of the analyte, as well as the degree of preservation of blood–retinal barrier integrity, which must be taken into account during result interpretation.	[91]
	Determination of rapidly metabolized substances (e.g., cocaine).	Low enzymatic activity, including the absence of pseudocholinesterase, limits the degradation of compounds susceptible to rapid metabolism, thereby enhancing their analytical stability.	Not all compounds exhibit the same ability to penetrate into the vitreous humor; therefore, interpretation of results requires knowledge of the pharmacokinetic properties of the analyzed substance.	[93,94]
Breast milk.	Analysis of cannabinoids in breast milk.	The high lipid content of breast milk and the lipophilic nature of cannabinoids promote their accumulation and facilitate analytical detection. This matrix demonstrates high utility for the determination of cannabis compounds and their metabolites.	Its application primarily concerns highly lipophilic compounds. The extent of penetration of other substances depends on their physicochemical properties.	[99,100]
	Ultra-high-performance liquid chromatography–tandem mass spectrometry (UPLC–MS/MS).	It is characterized by high analytical sensitivity, specificity, and selectivity for the determination of cannabinoids in breast milk. The method enables the detection of low analyte concentrations in a complex biological matrix.	The method requires highly specialized instrumentation, appropriately validated analytical procedures, and qualified laboratory personnel.	[101]
Placenta and umbilical cord tissue	Liquid chromatography–tandem mass spectrometry (LC–MS/MS)	A validated and effective method for the determination of a broad range of substances in placental and umbilical cord tissue samples. It enables the quantitative analysis of nicotine, cotinine, hydroxycotinine, and numerous psychoactive substances, including morphine, codeine, cocaine, benzoylecgonine, amphetamine, methamphetamine, methadone, and MDMA.	It requires advanced laboratory facilities, high-quality biological material, and appropriate validation of analytical procedures for individual analytes.	[106,107]
Oral fluid	Liquid chromatography with electrospray ionization coupled to tandem mass spectrometry (ESI-LC–MS/MS	A validated method with high sensitivity and accuracy, enabling the determination of testosterone levels in both serum and oral fluid. It may be applied in cases involving suspected abuse of anabolic–androgenic steroids.	It requires specialized analytical instrumentation and appropriate validation for specific analytes and concentration ranges.	[112]
	Analysis of drugs and sedative–hypnotic agents in oral fluid (LC–MS)	It enables the detection of psychoactive substances, including sedative–hypnotic agents such as zaleplon, zopiclone, and zolpidem, in both clinical and postmortem investigations.	The diagnostic utility depends on the timing of sample collection, the pharmacokinetic properties of the substance, and the extent of its transfer into oral fluid.	[113]
evidence materials	Hyperspectral imaging (HSI—Hyperspectral Imaging))	A modern, contactless, and non-destructive technique enabling the simultaneous analysis of spectral and spatial information. It allows for the identification, localization, and characterization of biological fluids, including blood, saliva, urine, semen, vaginal secretions, and menstrual blood. It demonstrates high sensitivity, enabling the detection of highly diluted and partially obscured traces. The technique preserves sample integrity for subsequent analyses.	It requires advanced instrumentation and specialized interpretation of spectral data. Its effectiveness may depend on the type of substrate, environmental conditions, and the degree of degradation of the examined material.	[119,120,121,122]

## Data Availability

No new data were created or analyzed in this study. Data sharing is not applicable to this article.

## Abbreviations

The following abbreviations are used in this manuscript:

TIAFT	The International Association of Forensic Toxicologists
STA	Systematic Toxicological Analysis
PMR	Postmortem redistribution
C/P	Cardiac Blood to Peripheral Blood Concentration
pKa	Acid dissociation constant
Vd	Volume of distribution
pH	potential of hydrogen
PMI	postmortem interval
RS	Raman spectroscopy
HPLC	High-Performance Liquid Chromatography
GC–MS	gas chromatography–mass spectrometry
LC–MS/MS	liquid chromatography coupled with tandem mass spectrometry
GC–MS/MS	gas chromatography–tandem mass spectrometry
GHB	gamma-hydroxybutyric acid
GC	gas chromatography
GC–FID	gas chromatography with flame ionization detection
NaNO_2_	sodium nitrite
MDMA	3,4-methylenedioxymethamphetamine
PAR	pulp/tooth area ratio
AAR	aspartic acid racemization
UV	ultraviolet
CSF	cerebrospinal fluid
PTMSiK	Polish Society of Forensic Medicine and Criminology
r	Pearson correlation coefficient
p	probability/statistical significance
MRI	magnetic resonance imaging
CT	computed tomography
BRB	blood–retinal barier
WHO	World Health Organization
UPLC	MS/MS ultra-performance liquid chromatography coupled with tandem mass spectrometry
ESI-LC	MS/MS electrospray ionization liquid chromatography–tandem mass spectrometry
LC–MS	liquid chromatography coupled with mass spectrometry
HSI	hyperspectral imaging
miRNA	microRNA
ncRNA	non-coding RNA
mRNA	messenger RNA
MZ	monozygotic
qRT-PCR	quantitative real-time PCR
DNA	deoxyribonucleic acid
CUD	Cocaine use disorder
DNAm	DNA methylation
DMR	differentially methylated regions
BA9	Brodmann area 9
GRIK2	glutamate ionotropic receptor kainate type subunit 2
BEGAIN	Brain-Enriched Guanylate Kinase-Associated Protein
BDNF	Brain-derived neurotrophic factor
TrkB	Tropomyosin receptor kinase B
AUD	alcohol use disorder
AH	human alcoholic hepatitis
TNF	tumour necrosis factor
Fn14	Fibroblast growth factor-inducible 14
FASE	The Forensic Anthropology Society of Europe
OSAC	The Organization of Scientific Area Committees for Forensic Science

## References

[1] Manousi N., Samanidou V. (2021). Green sample preparation of alternative biosamples in forensic toxicology. Sustain. Chem. Pharm..

[2] Reihani A., Marboutian F., Aghebat–bekheir S., Reyhani A., Akhgari M. (2024). Diagnostic Aspects of Paraquat in the Forensic Toxicology: A Systematic Review. Acad. Forensic Pathol..

[3] Kishikawa N., Higuchi S., Ohyama K., Nakashima K., Kuroda N. (2013). A simple and rapid chemiluminescence assay for on-site analysis of paraquat using a portable luminometer. Forensic Toxicol..

[4] Turkmen Z., Zengin S., Kuloglu Genc M., Yayla M., Tekin Bulbul T., Mercan S. (2023). The Role of Forensic Veterinary Toxicology in Pet Custody Cases. J. Anal. Toxicol..

[5] Smith M.P., Bluth M.H. (2016). Forensic Toxicology. Clin. Lab. Med..

[6] Stimpfl T., Muller C., Gergov M., Lebeau M.A., Polettini A.E., Sporkert F., Weinmann W. (2014). TIAFT—The International Association of Forensic Toxicologists Committee of Systematic Toxicological Analysis. https://api.semanticscholar.org/CorpusID:39880003.

[7] Cengiz S., Ulukan Ö., Ates I., Tugcu H. (2006). Determination of morphine in postmortem rabbit bone marrow and comparison with blood morphine concentrations. Forensic Sci. Int..

[8] Vandenbosch M., Rooseleers L., Van Den Bogaert W., Wuestenbergs J., Van De Voorde W., Cuypers E. (2020). Skeletal tissue, a viable option in forensic toxicology? A view into post mortem cases. Forensic Sci. Int..

[9] Wiart J.F., Hakim F., Andry A., Eiden C., Drevin G., Lelièvre B., Rougé-Maillart C., Decourcelle M., Lemaire-Hurtel A.S., Allorge D. (2020). Pitfalls of toxicological investigations in hair, bones, and nails in extensively decomposed bodies: Illustration with two cases. Int. J. Leg. Med..

[10] Wiebe T.R., Watterson J.H. (2014). Analysis of tramadol and O -desmethyltramadol in decomposed skeletal tissues following acute and repeated tramadol exposure by gas chromatography mass spectrometry. Forensic Sci. Int..

[11] Abdelaal G.M.M., Hegazy N.I., Etewa R.L., Elmesallamy G.E.A. (2023). Postmortem redistribution of drugs: A literature review. Forensic Sci. Med. Pathol..

[12] Stephenson L., Van Den Heuvel C., Scott T., Byard R.W. (2024). Difficulties associated with the interpretation of postmortem toxicology. J. Anal. Toxicol..

[13] Sastre C., Bartoli C., Baillif-Couniou V., Leonetti G., Pelissier-Alicot A.L. (2018). Post Mortem Redistribution of Drugs: Current State of Knowledge. Curr. Pharm. Des..

[14] Pélissier-Alicot A.L., Gaulier J.M., Champsaur P., Marquet P. (2003). Mechanisms Underlying Postmortem Redistribution of Drugs: A Review. J. Anal. Toxicol..

[15] Kennedy M.C. (2010). Post-mortem drug concentrations. Intern. Med. J..

[16] Yarema M.C., Becker C.E. (2005). Key concepts in postmortem drug redistribution. Clin. Toxicol..

[17] Garneau B., Roy C., Motard J., Desharnais B., Bouchard C., Mireault P. (2024). Atypical postmortem redistribution in chronic methadone consumers. J. Anal. Toxicol..

[18] Secco L., Palumbi S., Padalino P., Grosso E., Perilli M., Casonato M., Cecchetto G., Viel G. (2025). “Omics” and Postmortem Interval Estimation: A Systematic Review. Int. J. Mol. Sci..

[19] Javan G.T., Finley S.J., Can I., Wilkinson J.E., Hanson J.D., Tarone A.M. (2016). Human Thanatomicrobiome Succession and Time Since Death. Sci. Rep..

[20] Adserias-Garriga J., Hernández M., Quijada N.M., Rodríguez Lázaro D., Steadman D., Garcia-Gil J. (2017). Daily thanatomicrobiome changes in soil as an approach of postmortem interval estimation: An ecological perspective. Forensic Sci. Int..

[21] Javan G.T., Finley S.J., Abidin Z., Mulle J.G. (2016). The Thanatomicrobiome: A Missing Piece of the Microbial Puzzle of Death. Front. Microbiol..

[22] Woess C., Huck C.W., Badzoka J., Kappacher C., Arora R., Lindtner R.A., Zelger P., Schirmer M., Rabl W., Pallua J. (2023). Raman spectroscopy for postmortem interval estimation of human skeletal remains: A scoping review. J. Biophotonics.

[23] Huang W., Zhao S., Liu H., Pan M., Dong H. (2024). The Role of Protein Degradation in Estimation Postmortem Interval and Confirmation of Cause of Death in Forensic Pathology: A Literature Review. Int. J. Mol. Sci..

[24] Shan X., Cao C., Yang B. (2022). Analytical Approaches for the Determination of Buprenorphine, Methadone and Their Metabolites in Biological Matrices. Molecules.

[25] Shi Y., Cui X., Shen M., Xiang P. (2016). Quantitative analysis of the endogenous GHB level in the hair of the Chinese population using GC/MS/MS. J. Forensic Leg. Med..

[26] Leung K.W., Wong Z.C.F., Ho J.Y.M., Yip A.W.S., Ng J.S.C., Ip S.P.H., Ng W.Y.Y., Ho K.K.L., Duan R., Zhu K.Y. (2016). Determination of hair ketamine cut-off value from Hong Kong ketamine users by LC–MS/MS analysis. Forensic Sci. Int..

[27] Baeck S., Han E., Chung H., Pyo M. (2011). Effects of repeated hair washing and a single hair dyeing on concentrations of methamphetamine and amphetamine in human hairs. Forensic Sci. Int..

[28] Bertol E., Argo A., Procaccianti P., Vaiano F., Di Milia M.G., Furlanetto S., Mari F. (2012). Detection of gamma-hydroxybutyrate in hair: Validation of GC–MS and LC–MS/MS methods and application to a real case. J. Pharm. Biomed. Anal..

[29] Lendoiro E., Quintela Ó., De Castro A., Cruz A., López-Rivadulla M., Concheiro M. (2012). Target screening and confirmation of 35 licit and illicit drugs and metabolites in hair by LC–MSMS. Forensic Sci. Int..

[30] Scanferla D.T.P., Sano Lini R., Marchioni C., Mossini S.A.G. (2022). Drugs of abuse: A narrative review of recent trends in biological sample preparation and chromatographic techniques. Forensic Chem..

[31] Yang H., Liu C., Zhu C., Zheng Y., Li J., Zhu Q., Wang H., Fang X., Liu Q., Liang M. (2023). Determination of ten antipsychotics in blood, hair and nails: Validation of a LC–MS/MS method and forensic application of keratinized matrix analysis. J. Pharm. Biomed. Anal..

[32] Xiang P., Shen M., Drummer O.H. (2015). Review: Drug concentrations in hair and their relevance in drug facilitated crimes. J. Forensic Leg. Med..

[33] Klausz G., Kass K., Sótonyi P., Róna K. (2006). Hair analysis of abused and therapeutic drugs in forensic toxicology. Orv. Hetil..

[34] Kintz P. (2012). Segmental hair analysis can demonstrate external contamination in postmortem cases. Forensic Sci. Int..

[35] Küting T., Madea B., Hess C., Krämer M. (2022). Comparative Study: Postmortem Long-Term Stability of Endogenous GHB in Cardiac Blood, Femoral Blood, Vitreous Humor, Cerebrospinal Fluid and Urine with and without Sodium Fluoride Stabilization. J. Anal. Toxicol..

[36] Ji J.J., Xu D., Yan H., Xiang P., Shen M. (2023). Micro-segmental analysis of the entry pathway and distribution of zolpidem in hair from different scalp regions after a single dose. Front. Chem..

[37] Krumbiegel F., Hastedt M., Westendorf L., Niebel A., Methling M., Parr M.K., Tsokos M. (2016). The use of nails as an alternative matrix for the long-term detection of previous drug intake: Validation of sensitive UHPLC-MS/MS methods for the quantification of 76 substances and comparison of analytical results for drugs in nail and hair samples. Forensic Sci. Med. Pathol..

[38] Solimini R., Minutillo A., Kyriakou C., Pichini S., Pacifici R., Busardo F.P. (2018). Nails in Forensic Toxicology: An Update. Curr. Pharm. Des..

[39] Palmeri A., Pichini S., Pacifici R., Zuccaro P., Lopez A. (2000). Drugs in Nails: Physiology, Pharmacokinetics and Forensic Toxicology. Clin. Pharmacokinet..

[40] Baumgartner M.R. (2014). Nails: An Adequate Alternative Matrix in Forensic Toxicology for Drug Analysis?. Bioanalysis.

[41] Cobo-Golpe M., de-Castro-Ríos A., Cruz A., López-Rivadulla M., Lendoiro E. (2021). Determination and Distribution of Cannabinoids in Nail and Hair Samples. J. Anal. Toxicol..

[42] Raikos N., Tsoukali H., Njau S.N. (2001). Determination of opiates in postmortem bone and bone marrow. Forensic Sci. Int..

[43] Vandenbosch M., Somers T., Cuypers E. (2018). Distribution of Methadone and Metabolites in Skeletal Tissue. J. Anal. Toxicol..

[44] Emmons A.L., Mundorff A.Z., Keenan S.W., Davoren J., Andronowski J., Carter D.O., DeBruyn J.M. (2020). Characterizing the postmortem human bone microbiome from surface-decomposed remains. PLoS ONE.

[45] Ortiz-Herrero L., Uribe B., Armas L.H., Alonso M.L., Sarmiento A., Irurita J., Alonso R.M., Maguregui M.I., Etxeberria F., Bartolomé L. (2021). Estimation of the post-mortem interval of human skeletal remains using Raman spectroscopy and chemometrics. Forensic Sci. Int..

[46] Fernandez-Lopez L., Mancini R., Pellegrini M., Concetta Rotolo M., Luna A., Falcon M. (2020). Postmortem analysis of quetiapine and pregabalin in human bone. Leg. Med..

[47] Franceschetti L., Di Candia D., Giordano G., Carabelli I., Vignali G., Cattaneo C. (2021). Drugs in bone: Detectability of substances of toxicological interest in different states of preservation. J. Forensic Sci..

[48] Giordano G., Biehler-Gomez L., Seneci P., Cattaneo C., Di Candia D. (2021). Detecting drugs in dry bone: A pilot study of skeletal remains with a post-mortem interval over 23 years. Int. J. Leg. Med..

[49] Fernandez-Lopez L., Pellegrini M., Rotolo M.C., Luna Maldonado A., Falcon M., Mancini R. (2019). Development and validation of a method for analysing of duloxetine, venlafaxine and amitriptyline in human bone. Forensic Sci. Int..

[50] Vardakou I., Athanaselis S., Pistos C., Papadodima S., Spiliopoulou C., Moraitis K. (2014). The clavicle bone as an alternative matrix in forensic toxicological analysis. J. Forensic Leg. Med..

[51] Márquez-Grant N., Baldini E., Jeynes V., Biehler-Gomez L., Aoukhiyad L., Passalacqua N.V., Giordano G., Di Candia D., Cattaneo C. (2022). How Do Drugs Affect the Skeleton? Implications for Forensic Anthropology. Biology.

[52] Mancini R., Fernadez-Lopez L., Falcon M., Pellegrini M., Luna A., Rotolo M. (2021). Postmortem Analysis of Benzodiazepines in Human Bone by Gas Chromatography–Mass Spectrometry. J. Anal. Toxicol..

[53] López-Rabuñal A., Lendoiro E., Concheiro M., López-Rivadulla M., Cruz A., de-Castro-Ríos A. (2020). LC–MS-MS Method for the Determination of Antidepressants and Benzodiazepines in Meconium. J. Anal. Toxicol..

[54] Henning K., Dziadosz M., Klintschar M., Piep B., Teske J. (2018). Analyzing histological material to determine ajmaline and other drugs using high-performance liquid chromatography/tandem mass spectrometry. Drug Test. Anal..

[55] Behnoush B., Sheikhazadi A., Bazmi E., Fattahi A., Sheikhazadi E., Saberi Anary S.H. (2015). Comparison of UHPLC and HPLC in Benzodiazepines Analysis of Postmortem Samples: A Case–Control Study. Medicine.

[56] Sebo Z.L., Rendina-Ruedy E., Ables G.P., Lindskog D.M., Rodeheffer M.S., Fazeli P.K., Horowitz M.C. (2019). Bone Marrow Adiposity: Basic and Clinical Implications. Endocr. Rev..

[57] Wietecha-Posłuszny R., Lendor S., Garnysz M., Zawadzki M., Kościelniak P. (2017). Human bone marrow as a tissue in post-mortem identification and determination of psychoactive Substances—Screening methodology. J. Chromatogr. B.

[58] Chiarilli M.G., Delli Pizzi A., Mastrodicasa D., Febo M.P., Cardinali B., Consorte B., Cifaratti A., Panara V., Caulo M., Cannataro G. (2021). Bone marrow magnetic resonance imaging: Physiologic and pathologic findings that radiologist should know. Radiol. Med..

[59] Marinelli Busilacchi E., Morsia E., Poloni A. (2024). Bone Marrow Adipose Tissue. Cells.

[60] Wheeler A., Czado N., Gangitano D., Turnbough M., Hughes-Stamm S. (2017). Comparison of DNA yield and STR success rates from different tissues in embalmed bodies. Int. J. Leg. Med..

[61] Laudani A., Sangregorio D., Zuccarello P., Licciardello T., Grasso S., Romano G., Barbera N. (2021). Development and Validation of a Method for the Detection and Quantification of Antidepressants and Antipsychotics in Bone Samples by GC–MS. J. Anal. Toxicol..

[62] Orfanidis A., Gika H., Mastrogianni O., Krokos A., Theodoridis G., Zaggelidou E., Raikos N. (2018). Determination of drugs of abuse and pharmaceuticals in skeletal tissue by UHPLC–MS/MS. Forensic Sci. Int..

[63] Cvetko M., Knific T., Frangež R., Motaln H., Rogelj B., Alibegović A., Gombač M. (2024). Postmortem chondrocyte viability in porcine articular cartilage: Influence of time, temperature, and burial under winter conditions. J. Forensic Sci..

[64] Tomsia M., Chełmecka E., Głaz M., Nowicka J. (2023). Epiglottis Cartilage, Costal Cartilage, and Intervertebral Disc Cartilage as Alternative Materials in the Postmortem Diagnosis of Methanol Poisoning. Toxics.

[65] Tomsia M., Nowicka J., Skowronek R., Woś M., Wójcik J., Droździok K., Zorychta M., Javan G.T., Chełmecka E. (2020). A Comparative Study of Ethanol Concentration in Costal Cartilage in Relation to Blood and Urine. Processes.

[66] Tomsia M., Głaz M., Nowicka J., Szczepański M. (2021). Sodium nitrite detection in costal cartilage and vitreous humor - Case report of fatal poisoning with sodium nitrite. J. Forensic Leg. Med..

[67] Durão C., Dinis-Oliveira R.J. (2021). Comment on Tomsia M. et al. Article “sodium nitrite detection in costal cartilage and vitreous humor—Case report of fatal poisoning with sodium nitrite”. J. Forensic Leg. Med..

[68] Tomsia M., Droździok K., Javan G.T., Skowronek R., Szczepański M., Chełmecka E. (2021). Costal cartilage ensures low degradation of DNA needed for genetic identification of human remains retrieved at different decomposition stages and different postmortem intervals. Postępy Hig. I Med. Dośw..

[69] Meng H., Zhang M., Xiao B., Chen X., Yan J., Zhao Z., Ma K., Shen Y., Xie J. (2019). Forensic age estimation based on the pigmentation in the costal cartilage from human mortal remains. Leg. Med..

[70] Riedel A., Neukamm M.A., Klima M., Henkel K., Auwärter V., Altenburger M.J. (2024). Drugs in dental biofilm and enamel—A pilot study. Heliyon.

[71] Jakubovics N.S., Goodman S.D., Mashburn-Warren L., Stafford G.P., Cieplik F. (2021). The dental plaque biofilm matrix. Periodontol. 2000.

[72] Henkel K., Klima M., Auwärter V., Altenburger M.J., Neukamm M.A. (2022). Dental Plaque Concentrations of Methadone, Morphine and Their Metabolites in Opioid Replacement Therapy and in Postmortem Cases. J. Anal. Toxicol..

[73] Marsh P.D. (2006). Dental plaque as a biofilm and a microbial community—Implications for health and disease. BMC Oral Health.

[74] Klima M., Altenburger M.J., Kempf J., Auwärter V., Neukamm M.A. (2016). Determination of medicinal and illicit drugs in post mortem dental hard tissues and comparison with analytical results for body fluids and hair samples. Forensic Sci. Int..

[75] Bianchi I., Croce E., Gelli F., Dimitrova A., Pradella F., Defraia B., Capasso E., Vaiano F., Mannaioni G., Pinchi V. (2025). A Pilot Study on the Deposition of Drugs in Dental Tissues as Alternative Matrices for Forensic Toxicology. Acta Stomatol Croat..

[76] Braga S., Caldas I.M., Dinis-Oliveira R.J. (2025). Forensic significance of postmortem pink teeth: A narrative review. Arch. Oral Biol..

[77] Minegishi S., Utsuno H., Ohta J., Namiki S., Toya M., Sumi N., Unuma K., Saitoh H., Iwase H., Uemura K. (2022). Sixty-eight cases of postmortem pink teeth observed in dental autopsies of unidentified cadavers. J. Forensic Sci..

[78] Kotze D., Mole C.G., Phillips V.M., Gibbon V.E. (2025). Exploring optimal methods for age-at-death estimation using pulp/tooth area ratios: A South African study. Int. J. Leg. Med..

[79] Teglind R., Dawidson I., Balkefors J., Alkass K. (2021). Analysis of 14C, 13C and Aspartic Acid Racemization in Teeth and Bones to Facilitate Identification of Unknown Human Remains: Outcomes of Practical Casework. Biomolecules.

[80] Sears V.G., Bleay S.M., Bandey H.L., Bowman V.J. (2012). A methodology for finger mark research. Sci. Justice.

[81] Girod A., Ramotowski R., Weyermann C. (2012). Composition of fingermark residue: A qualitative and quantitative review. Forensic Sci. Int..

[82] Mink T., Voorhaar A., Stoel R., De Puit M. (2013). Determination of efficacy of fingermark enhancement reagents; the use of propyl chloroformate for the derivatization of fingerprint amino acids extracted from paper. Sci. Justice.

[83] Cadd S., Islam M., Manson P., Bleay S. (2015). Fingerprint composition and aging: A literature review. Sci. Justice.

[84] Adamowicz P., Bigosińska J., Gil D., Suchan M., Tokarczyk B. (2024). Drugs detection in fingerprints. J. Pharm. Biomed. Anal..

[85] Gomes F.M., De Pereira C.M.P., De Cássia Mariotti K., Magaiver Pereira T., Dos Santos N.A., Romão W. (2023). Study of latent fingerprints—A review. Forensic Chem..

[86] Dinis-Oliveira R.J., Vieira D.N., Magalhães T. (2016). Guidelines for Collection of Biological Samples for Clinical and Forensic Toxicological Analysis. Forensic Sci. Res..

[87] Wachholz P., Skowronek R., Pawlas N. (2021). Cerebrospinal fluid in forensic toxicology: Current status and future perspectives. J. Forensic Leg. Med..

[88] Wachholz P., Skowronek R., Pawlas N. (2022). Assessing the applicability of cerebrospinal fluid collected from the spinal cord for the determination of ethyl alcohol in post-mortem toxicology. Forensic Sci. Med. Pathol..

[89] De-Giorgio F., Ciasca G., Fecondo G., Mazzini A., De Spirito M., Pascali V.L. (2021). Estimation of the time of death by measuring the variation of lateral cerebral ventricle volume and cerebrospinal fluid radiodensity using postmortem computed tomography. Int. J. Leg. Med..

[90] Trella S., Reinert C., Heinsen H., Preiß U., Monoranu C., Zwirner J., Ondruschka B., Bohnert M., Bohnert S. (2024). The polychromatism of postmortem cerebrospinal fluid. Forensic Sci. Med. Pathol..

[91] Kugelberg F.C., Jones A.W. (2007). Interpreting results of ethanol analysis in postmortem specimens: A review of the literature. Forensic Sci. Int..

[92] Moschovakou D., Ntoupa S.P., Dona A., Athanaselis S., Spiliopoulou C., Nikolaou P., Papoutsis I. (2024). Vitreous humor in the forensic toxicology of quetiapine and its metabolites. Forensic Toxicol..

[93] Wójtowicz A., Reciak M., Nowak P.M., Wietecha-Posłuszny R. (2022). A sustainable approach for the stability study of psychotropic substances using vitreous humor and liver as alternative matrices. Anal. Bioanal. Chem..

[94] Margalho C., Castanheira A., Real F.C., Gallardo E., López-Rivadulla M. (2016). Determination of “new psychoactive substances” in postmortem matrices using microwave derivatization and gas chromatography–mass spectrometry. J. Chromatogr. B..

[95] Ramos Santos W.J., De Martinis B.S. (2020). Psychoactive Substances in Human Breast Milk: A Review of Analytical Strategies for their Investigation. Bioanalysis.

[96] Strom K., Jarzynka S., Minkiewicz-Zochniak A., Barbarska O., Olędzka G., Wesolowska A. (2022). Microbiological Quality of Milk Donated to the Regional Human Milk Bank in Warsaw in the First Four Years of Activity. Healthcare.

[97] Çelik E., Cemali Ö., Şahin T.Ö., Deveci G., Biçer N.Ç., Hirfanoğlu İ.M., Ağagündüz D., Budán F. (2024). Human Breast Milk Exosomes: Affecting Factors, Their Possible Health Outcomes, and Future Directions in Dietetics. Nutrients.

[98] Lyons K.E., Ryan C.A., Dempsey E.M., Ross R.P., Stanton C. (2020). Breast Milk, a Source of Beneficial Microbes and Associated Benefits for Infant Health. Nutrients.

[99] De Campos E.G., Da Costa B.R.B., Dos Santos F.S., Monedeiro F., Alves M.N.R., Santos Junior W.J.R., De Martinis B.S. (2022). Alternative matrices in forensic toxicology: A critical review. Forensic Toxicol..

[100] Motas M., Jiménez S., Oliva J., Cámara M.Á., Pérez-Cárceles M.D. (2021). Heavy Metals and Trace Elements in Human Breast Milk from Industrial/Mining and Agricultural Zones of Southeastern Spain. Int. J. Environ. Res. Public Health.

[101] Ramnarine R.S., Poklis J.L., Wolf C.E. (2019). Determination of Cannabinoids in Breast Milk Using QuEChERS and Ultra-Performance Liquid Chromatography and Tandem Mass Spectrometry. J. Anal. Toxicol..

[102] Moore C., Negrusz N. (1995). Drugs of Abuse in Meconium. Forensic Sci. Rev..

[103] Palmer K.L., Krasowski M.D., Langman L.J., Snozek C.L.H. (2019). Alternate Matrices: Meconium, Cord Tissue, Hair, and Oral Fluid. LC-MS in Drug Analysis.

[104] Colby J.M. (2017). Comparison of umbilical cord tissue and meconium for the confirmation of in utero drug exposure. Clin. Biochem..

[105] Moore C., Negrusz A., Lewis D. (1998). Determination of drugs of abuse in meconium. J. Chromatogr. B Biomed. Sci. Appl..

[106] Paniagua-González L., Jiménez-Morigosa C., Lendoiro E., Concheiro M., Cruz A., López-Rivadulla M., De Castro A. (2018). Development and validation of a liquid chromatography–tandem mass spectrometry method for the determination of nicotine and its metabolites in placenta and umbilical cord. Drug Test. Anal..

[107] De Castro A., Díaz A., Piñeiro B., Lendoiro E., Cruz A., López-Rivadulla M., Concheiro M. (2013). Simultaneous determination of opiates, methadone, amphetamines, cocaine, and metabolites in human placenta and umbilical cord by LC-MS/MS. Anal. Bioanal. Chem..

[108] Carlier J., La Maida N., Di Trana A., Huestis M.A., Pichini S., Busardò F.P. (2020). Testing Unconventional Matrices to Monitor for Prenatal Exposure to Heroin, Cocaine, Amphetamines, Synthetic Cathinones, and Synthetic Opioids. Ther. Drug Monit..

[109] Proctor G.B., Shaalan A.M. (2021). Disease-Induced Changes in Salivary Gland Function and the Composition of Saliva. J. Dent. Res..

[110] Costa B.R.B.D., Santos Júnior W.J.R., Maximiano I.F., Gomes N.C., Freitas B.T., De Martinis B.S. (2021). Application of Microextraction Techniques in Alternative Biological Matrices with Focus on Forensic Toxicology: A Review. Bioanalysis.

[111] Sprega G., Di Giorgi A., Poyatos L., Papaseit E., Pérez-Mañá C., Tini A., Pichini S., Busardò F.P., Faro A.F.L., Farré M. (2023). Usefulness of Oral Fluid for Measurement of Methylone and Its Metabolites: Correlation with Plasma Drug Concentrations and the Effect of Oral Fluid pH. Metabolites.

[112] Lood Y., Aardal E., Ahlner J., Ärlemalm A., Carlsson B., Ekman B., Wahlberg J., Josefsson M. (2021). Determination of testosterone in serum and saliva by liquid chromatography-tandem mass spectrometry: An accurate and sensitive method applied on clinical and forensic samples. J. Pharm. Biomed. Anal..

[113] Zhang Y.Z., He H.Y., She C.M., Lian J. (2015). Research Progress on Forensic Toxicology of Z-drugs. Fa Yi Xue Za Zhi.

[114] Truver M.T., Palmquist K.B., Swortwood M.J. (2019). Oral Fluid and Drug Impairment: Pairing Toxicology with Drug Recognition Expert Observations. J. Anal. Toxicol..

[115] Aslan R., Aydoğdu M., Bostancı H.İ., Ertaş H., Akgür S.A. (2023). Development of analytical method for illegal substances in sweat and comparison of the effectiveness of sweat collection materials. Leg. Med..

[116] Di Giorgi A., Sprega G., Poyatos L., Papaseit E., Pérez-Mañá C., Di Trana A., Varì M.R., Busardò F.P., Pichini S., Zaami S. (2023). Sweat Testing for the Detection of Methylone after Controlled Administrations in Humans. Int. J. Mol. Sci..

[117] Tavares L., Monedeiro F., Bordin D.M., De Martinis B.S. (2020). Investigation of Ayahuasca β-Carboline Alkaloids and Tryptamine in Sweat Samples from Religious Community Participants by GC-MS. J. Anal. Toxicol..

[118] Schneider T.D., Kraemer T., Steuer A.E. (2023). Untargeted Metabolomics Profiling for Determination of the Time since Deposition of Biofluids in a Forensic Context: A Proof-of-Concept for Urine, Saliva, and Semen in Addition to Blood. Anal. Chem..

[119] Pradeep A.S., Babu J., Sudaroli Sandana J., Deivalakshmi S. (2024). Innovations in forensic science: Comprehensive review of hyperspectral imaging for bodily fluid analysis. Forensic Sci. Int..

[120] Mariotti K.D.C., Ortiz R.S., Ferrão M.F. (2023). Hyperspectral imaging in forensic science: An overview of major application areas. Sci. Justice.

[121] Pallocci M., Treglia M., Passalacqua P., Luca L.D., Zanovello C., Mazzuca D., Guarna F., Gratteri S., Marsella L.T. (2022). Forensic applications of hyperspectral imaging technique: A narrative review. Med. Leg. J..

[122] Crowther M., Li B., Thompson T., Islam M. (2021). A comparison between visible wavelength hyperspectral imaging and digital photography for the detection and identification of bloodstained footwear marks. J. Forensic Sci..

[123] Giorgetti A., Pelletti G., Fais P., Giovannini E., Barone R., Pelotti S., Pascali J.P. (2022). The use of fly artifacts in a crime scene: Is there any application for forensic toxicology?. J. Forensic Sci..

[124] Cano-Trujillo C., García-Ruiz C., Ortega-Ojeda F.E., Romolo F., Montalvo G. (2023). Forensic analysis of biological fluid stains on substrates by spectroscopic approaches and chemometrics: A review. Anal. Chim. Acta..

[125] Nikolaou P., Papoutsis I., Dona A., Spiliopoulou C., Athanaselis S. (2013). Toxicological analysis of formalin-fixed or embalmed tissues: A review. Forensic Sci. Int..

[126] Takayasu T. (2013). Toxicological Analyses of Medications and Chemicals in Formalin-Fixed Tissues and Formalin Solutions: A Review. J. Anal. Toxicol..

[127] Uekusa K., Hayashida M., Ohno Y. (2015). Forensic toxicological analyses of drugs in tissues in formalin solutions and in fixatives. Forensic Sci. Int..

[128] Tzatzarakis M.N., Alegakis A.K., Kavvalakis M.P., Vakonaki E., Stivaktakis P.D., Kanaki K., Vardavas A.I., Barbounis E.G., Tsatsakis A.M. (2016). Comparative Evaluation of Drug Deposition in Hair Samples Collected from Different Anatomical Body Sites. J. Anal. Toxicol..

[129] Yan H., Xiang P., Shen M. (2021). Current status of hair analysis in forensic toxicology in China. Forensic Sci. Res..

[130] Miolo G., Tucci M., Menilli L., Stocchero G., Vogliardi S., Scrivano S., Montisci M., Favretto D. (2018). A Study on Photostability of Amphetamines and Ketamine in Hair Irradiated under Artificial Sunlight. Brain Sci..

[131] Rygaard K., Linnet K., Johansen S.S. (2021). A Systematic Review of Metabolite-to-Drug Ratios of Pharmaceuticals in Hair for Forensic Investigations. Metabolites.

[132] Magalhães T.P., Cravo S., Silva D.D.D., Dinis-Oliveira R.J., Afonso C., Lourdes Bastos M.D., Carmo H. (2018). Quantification of Methadone and Main Metabolites in Nails. J. Anal. Toxicol..

[133] Kuwayama K., Miyaguchi H., Kanamori T., Tsujikawa K., Yamamuro T., Segawa H., Okada Y., Iwata Y.T. (2022). Micro-segmental hair analysis: Detailed procedures and applications in forensic toxicology. Forensic Toxicol..

[134] Zhuo Y., Xiang P., Wu J., Wang X. (2022). Segmental hair analysis for flunitrazepam and 7-aminoflunitrazepam in users: A comparison to existing literature. Forensic Sci. Res..

[135] Takahashi S., Takada A., Saito K., Hara M., Yoneyama K., Nakanishi H. (2022). Diagnostic significance of the histopathology of bone marrow macrophages in forensic autopsies. Leg. Med..

[136] Sakr M.F., El-Khalek A.M.A., Mohammad N.S., Abouhashem N.S., Gaballah M.H., Ragab H.M. (2023). Estimation of postmortem interval using histological and oxidative biomarkers in human bone marrow. Forensic Sci. Med. Pathol..

[137] Cabarcos-Fernández P., Álvarez-Freire I., Rubio N.C., Bermejo-Barrera A.M., Moreda-Piñeiro A., Sánchez-Sellero I., Tabernero-Duque M.J. (2024). Evaluation of an Oral Fluid Collection Device and a Solid-Phase Extraction Method for the Determination of Coca Leaf Alkaloids by Gas Chromatography–Mass Spectrometry. Molecules.

[138] Liu Y., He H., Xiao Z.X., Ji A., Ye J., Sun Q., Cao Y. (2021). A systematic analysis of miRNA markers and classification algorithms for forensic body fluid identification. Brief. Bioinform..

[139] Bernini Di Michele A., Onofri V., Pesaresi M., Turchi C. (2023). The Role of miRNA Expression Profile in Sudden Cardiac Death Cases. Genes.

[140] Silva S.S., Lopes C., Teixeira A.L., Sousa M.J.C.D., Medeiros R. (2015). Forensic miRNA: Potential biomarker for body fluids?. Forensic Sci. Int. Genet..

[141] Chen X., Xu H., Lin Y., Zhu B. (2024). Forensic stability evaluation of selected miRNA and circRNA markers in human bloodstained samples exposed to different environmental conditions. Forensic Sci. Int..

[142] Li Y., Wang Z., Ishmael D., Lvy Y. (2023). The potential of using non-coding RNAs in forensic science applications. Forensic Sci. Res..

[143] Chen X., Xu H., Zhu B. (2023). Forensic validation of a combined analysis of mRNA and miRNA markers for precise tissue origin inferences of five kinds of body fluids by RT-qPCR. Electrophoresis.

[144] Fujimoto S., Manabe S., Morimoto C., Ozeki M., Hamano Y., Tamaki K. (2018). Optimal small-molecular reference RNA for RT-qPCR-based body fluid identification. Forensic Sci. Int. Genet..

[145] Glynn C.L. (2020). Potential applications of microRNA profiling to forensic investigations. RNA.

[146] Xiao C., Pan C., Liu E., He H., Liu C., Huang Y., Yi S., Huang D. (2019). Differences of microRNA expression profiles between monozygotic twins’ blood samples. Forensic Sci. Int. Genet..

[147] Grimm S.L., Mendez E.F., Stertz L., Meyer T.D., Fries G.R., Gandhi T., Kanchi R., Selvaraj S., Teixeira A.L., Kosten T.R. (2023). MicroRNA–mRNA networks are dysregulated in opioid use disorder postmortem brain: Further evidence for opioid-induced neurovascular alterations. Front. Psychiatry.

[148] O’Connell G.C., Smothers C.G., Winkelman C. (2020). Bioinformatic analysis of brain-specific miRNAs for identification of candidate traumatic brain injury blood biomarkers. Brain Inj..

[149] Gerra M.C., Dallabona C., Cecchi R. (2024). Epigenetic analyses in forensic medicine: Future and challenges. Int. J. Leg. Med..

[150] Dai E., Yu X., Zhang Y., Meng F., Wang S., Liu X., Liu D., Wang J., Li X., Jiang W. (2014). EpimiR: A database of curated mutual regulation between miRNAs and epigenetic modifications. Database.

[151] Vidaki A., Kayser M. (2018). Recent progress, methods and perspectives in forensic epigenetics. Forensic Sci. Int. Genet..

[152] Zumbrun E.E., Sido J.M., Nagarkatti P.S., Nagarkatti M. (2015). Epigenetic Regulation of Immunological Alterations Following Prenatal Exposure to Marijuana Cannabinoids and its Long Term Consequences in Offspring. J. Neuroimmune Pharmacol..

[153] Hollander J.A., Im H.I., Amelio A.L., Kocerha J., Bali P., Lu Q., Willoughby D., Wahlestedt C., Conkright M.D., Kenny P.J. (2010). Striatal microRNA controls cocaine intake through CREB signalling. Nature.

[154] Fernàndez-Castillo N., Cabana-Domínguez J., Corominas R., Cormand B. (2022). Molecular genetics of cocaine use disorders in humans. Mol. Psychiatry.

[155] Zillich E., Belschner H., Avetyan D., Andrade-Brito D., Martínez-Magaña J.J., Frank J., Mechawar N., Turecki G., Cabana-Domínguez J., Fernàndez-Castillo N. (2024). Multi-omics profiling of DNA methylation and gene expression alterations in human cocaine use disorder. Transl. Psychiatry.

[156] Poisel E., Zillich L., Streit F., Frank J., Friske M.M., Foo J.C., Mechawar N., Turecki G., Hansson A.C., Nöthen M.M. (2023). DNA methylation in cocaine use disorder–An epigenome-wide approach in the human prefrontal cortex. Front. Psychiatry.

[157] Peedicayil J., Kumar A. (2018). Epigenetic Drugs for Mood Disorders. Progress in Molecular Biology and Translational Science.

[158] Dee G., Ryznar R., Dee C. (2023). Epigenetic Changes Associated with Different Types of Stressors and Suicide. Cells.

[159] Haghighi F., Xin Y., Chanrion B., O’Donnell A.H., Ge Y., Dwork A.J., Arango V., Mann J.J. (2014). Increased DNA methylation in the suicide brain. Dialogues Clin. Neurosci..

[160] Gatta E., Guidotti A., Saudagar V., Grayson D.R., Aspesi D., Pandey S.C., Pinna G. (2021). Epigenetic Regulation of GABAergic Neurotransmission and Neurosteroid Biosynthesis in Alcohol Use Disorder. Int. J. Neuropsychopharmacol..

[161] Morrow A.L., Boero G., Porcu P. (2020). A Rationale for Allopregnanolone Treatment of Alcohol Use Disorders: Basic and Clinical Studies. Alcohol. Clin. Exp. Res..

[162] Gatta E., Auta J., Gavin D.P., Bhaumik D.K., Grayson D.R., Pandey S.C., Guidotti A. (2017). Emerging Role of One-Carbon Metabolism and DNA Methylation Enrichment on δ-Containing GABAA Receptor Expression in the Cerebellum of Subjects with Alcohol Use Disorders (AUD). Int. J. Neuropsychopharmacol..

[163] Furuya S., Cichocki J.A., Konganti K., Dreval K., Uehara T., Katou Y., Fukushima H., Kono H., Pogribny I.P., Argemi J. (2019). Histopathological and Molecular Signatures of a Mouse Model of Acute-on-Chronic Alcoholic Liver Injury Demonstrate Concordance with Human Alcoholic Hepatitis. Toxicol. Sci..

[164] Affò S., Dominguez M., Lozano J.J., Sancho-Bru P., Rodrigo-Torres D., Morales-Ibanez O., Moreno M., Millán C., Loaeza-Del-Castillo A., Altamirano J. (2013). Transcriptome analysis identifies TNF superfamily receptors as potential therapeutic targets in alcoholic hepatitis. Gut.

[165] Cecchi R. (2010). Estimating wound age: Looking into the future. Int. J. Leg. Med..

[166] Butzbach D.M. (2010). The influence of putrefaction and sample storage on post-mortem toxicology results. Forensic Sci. Med. Pathol..

[167] De Boer H.H., Obertová Z., Cunha E., Adalian P., Baccino E., Fracasso T., Kranioti E., Lefévre P., Lynnerup N., Petaros A. (2020). Strengthening the role of forensic anthropology in personal identification: Position statement by the Board of the Forensic Anthropology Society of Europe (FASE). Forensic Sci. Int..

[168] Morrison G.S., Neumann C., Geoghegan P.H. (2020). Vacuous standards—Subversion of the OSAC standards-development process. Forensic Sci. Int. Synerg..

